# Transfer RNA‐Derived Small RNA‐5’tiRNAGln Drives HNRNPC Phase Separation to Promote Breast Cancer Bone Metastasis by Regulating IGF1R

**DOI:** 10.1002/advs.77974

**Published:** 2026-09-29

**Authors:** Bingnan Wang, Dongsong Liu, Xiaozhi Fan, Jiale Fan, Xuesi liu, Yu Wang, Zixin Wang, Mo Cheng, Ping Zhou, Wangjun Yan

**Affiliations:** ^1^ Department of Musculoskeletal Oncology Fudan University Shanghai Cancer Center Shanghai China; ^2^ Department of Oncology Shanghai Medical College Fudan University Shanghai China; ^3^ State Key Laboratory of Medical Neurobiology MOE Frontiers Center for Brain Science Institutes of Brain Science Fudan University Shanghai China; ^4^ Department of Physiology and Pathophysiology School of Basic Medical Sciences Fudan University Shanghai China

**Keywords:** 5’tiRNA‐Gln, HNRNPC, IGF1R, Phase separation, tRNA‐derived fragment

## Abstract

Transfer RNA‐derived small RNAs (tsRNAs) are emerging tumor regulators, yet their roles in breast cancer (BC) bone metastasis (BM) remain poorly understood. Here, we identify 5’tiRNA‐Gln as a key pro‐metastatic tsRNA markedly upregulated in BM tissues. Functionally, 5’tiRNA‐Gln robustly enhances BC cell migration, invasion, and osteoclast differentiation. Mechanistically, 5’tiRNA‐Gln directly binds to the RRM domain of heterogeneous nuclear ribonucleoprotein C (HNRNPC), a novel phase‐separating protein. Biophysically, 5’tiRNA‐Gln acts as a “molecular glue” to accelerate HNRNPC liquid‐liquid phase separation. These phase‐separated condensates preferentially bind to the coding sequence (CDS) region of *IGF1R* mRNA, increasing its stability and hyperactivating downstream ERK signaling. Clinically, HNRNPC is amplified in BM tissues and predicts poor prognosis. Silencing HNRNPC dramatically suppresses bone‐destructive lesions and tumor burden in vivo. In conclusion, our study uncovers a novel biophysical mechanism wherein a tsRNA regulates protein phase separation to drive tumor metastasis. The 5’tiRNA‐Gln/HNRNPC/IGF1R axis represents a promising biomarker and therapeutic target for BC bone metastasis.

## Graphical abstract

This study reveals that 5'tiRNA‐Gln interacts with hnRNPC to promote IGF1R liquid‐liquid phase separation, which drives ERK1/2 signaling activation, EMT, and breast cancer bone metastasis. These findings highlight a novel mechanism of breast cancer bone metastasis and potential therapeutic target in metastatic breast cancer.

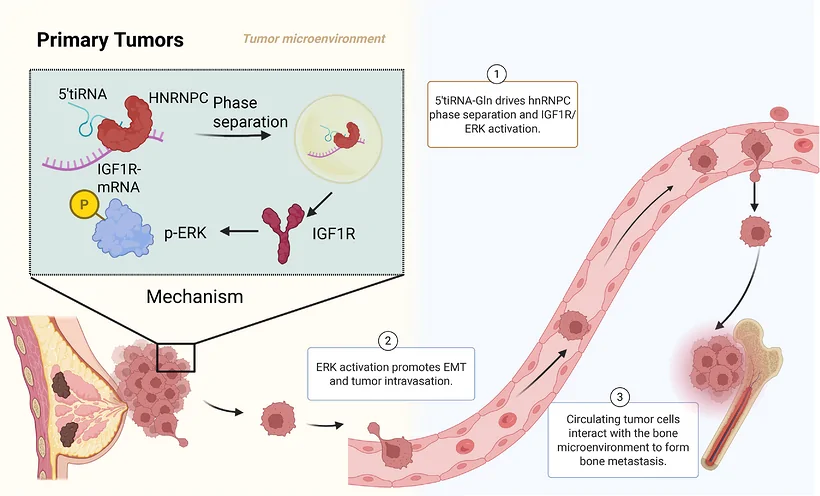

## 1 Introduction

Breast cancer remains the most prevalent malignancy among women and a leading cause of cancer‐related mortality globally [1]. Bone is the predominant site of distant metastasis in breast cancer, with approximately 65%–80% of patients with advanced disease eventually developing bone lesions [1, 2]. Clinically, bone metastasis (BM) precipitates severe skeletal‐related events (SREs)—including pathological fractures, hypercalcemia, spinal cord compression, and intractable bone pain—which drastically diminish patients’ quality of life [3]. Breast cancer metastasis is a multifaceted process governed by a complex interplay of intrinsic cellular factors and extrinsic microenvironmental cues. Upon extravasation, disseminated tumor cells (DTCs) frequently colonize the perivascular niche, where they engage in dynamic reciprocal crosstalk with various components of the bone marrow microenvironment, including osteoclasts, osteoblasts, immune cells, and stromal cells [4]. This bidirectional interaction establishes a notorious “vicious cycle”: tumor‐derived factors (such as RANKL, OPG, and PTHrP) stimulate osteoclast activation and bone resorption; concurrently, the resorbed bone matrix releases abundant growth factors (e.g., TGF‐β, BMPs) that further fuel tumor proliferation and survival [5, 6]. Notably, metastasis in the bone marrow niche is considered to be an important transfer station for further distant metastasis [7, 8]. Although osteoclast‐targeting therapies can alleviate skeletal complications and delay disease progression, they fail to substantially prolong overall survival in patients with advanced BM [9, 10]. Therefore, a deeper elucidation of the molecular mechanisms driving breast cancer bone metastasis is imperative to identify novel prognostic biomarkers and effective therapeutic targets.

Transfer RNA‐derived small RNAs (tsRNAs) are a class of non‐coding RNAs that exert important biological functions by regulating gene transcription and translation [11]. Accumulating evidence underscores the critical roles of tsRNAs in diverse physiological and pathological landscapes, particularly in tumor initiation, progression, and metastasis, highlighting their immense potential as diagnostic biomarkers and therapeutic entry points [12, 13, 14]. Based on their biogenesis and length, tsRNAs are broadly categorized into two major populations: tRNA‐derived stress‐induced small RNAs (tiRNAs) and tRNA‐derived fragments (tRFs). Under cellular stress, angiogenin (ANG) specifically cleaves the anticodon loop of mature tRNAs to generate tiRNA halves (29–50 nt). Conversely, shorter tRFs (16–28 nt) originate from precise processing of mature or precursor tRNAs, encompassing tRF‐5, tRF‐3, tRF‐2, tRF‐1, and i‐tRF according to their regional mapping [15]. Notably, previous studies have demonstrated that several tsRNAs, including tsRNA‐101, tsRNA‐53, and tsRNA‐26576, are highly expressed in breast cancer, and inhibition of these molecules significantly attenuates the invasive and metastatic capacity of breast cancer cells [16, 17, 18].

In this study, through transcriptomic profiling of tsRNA‐seq data from three pairs of primary breast tumors and matched bone metastatic lesions, we identified 5’tiRNA‐Gln as a prominently and specifically upregulated candidate in bone metastases. RNA in situ hybridization (ISH) confirmed that 5’tiRNA‐Gln expression is significantly elevated in bone‐metastatic breast cancer tissues compared with non‐metastatic primary tumors. Functional assays in vitro and in vivo demonstrated that 5’tiRNA‐Gln remarkably enhances the bone‐metastatic potential of breast cancer cells and potently induces osteoclast differentiation. Mechanistically, integrated analyses of RNA pull‐down, RIP‐seq, and eCLIP‐seq datasets revealed that 5’tiRNA‐Gln directly binds to the RNA recognition motif (RRM) domain of heterogeneous nuclear ribonucleoprotein C (HNRNPC). This binding promotes the liquid–liquid phase separation (LLPS) of HNRNPC, a process intrinsically driven by its intrinsically disordered region (IDR). Crucially, the 5’tiRNA‐Gln‐induced condensation of HNRNPC enhances its binding affinity toward the coding sequence (CDS) region of IGF1R mRNA, thereby stabilizing the transcript and boosting its expression to sustain the malignant phenotypes of breast cancer cells. Finally, therapeutic targeting of 5’tiRNA‐Gln successfully suppressed breast cancer bone metastasis in xenograft models. Collectively, our findings provide novel insights into the epigenetic landscape of breast cancer bone metastasis and highlight the 5’tiRNA‐Gln/HNRNPC/IGF1R axis as a promising diagnostic and therapeutic vulnerability for the intervention of bone metastasis.

## 2 Results

### 2.1 5’tiRNA‐Gln is Upregulated in Bone Metastatic Breast Cancer Lesions

To systematically investigate the expression profiles of tRNA‐derived small RNAs (tsRNAs) during breast cancer progression, we performed high‐throughput tsRNA sequencing on three primary breast cancer tissues and their corresponding patient‐matched bone metastatic lesions (Figure 1A). A total of 3,914 distinct tsRNA molecules were identified. Characterization of the tsRNA composition revealed that tRF‐5 was the most abundant subclass (1,595 species), followed by 5’tiRNAs (778 species) and tRF‐3 (709 species) (Figure S1A). Further analysis of parental tRNA origins indicated that the majority of these tsRNAs derived from tRNA^Cys–GCA^ (N = 207) (Figure S1B). Assessment of the length distribution showed that 20‐nt fragments constituted the most prevalent population, accounting for 686 distinct molecules (Figure S1C). Intriguingly, comparative expression analysis revealed that among the 3,914 identified tsRNAs, 26 were significantly upregulated and 3 were downregulated in bone metastases relative to primary lesions (Figure 1B). Notably, 5’tiRNA‐35^Gln–TTG–01^(hereafter designated as 5’tiRNA‐Gln) exhibited the most pronounced fold‐change and the highest statistical significance (Table S1). Interrogation of the MINTbase database [19] identified 5’tiRNA‐Gln as a fragment originating from the 5’ terminus (positions 1–35) of tRNA^Gln–TTG^, which maps to chromosomes 6 and 17 and adopts a characteristic stem‐loop secondary structure (Figure 1C,D).

**FIGURE 1:**
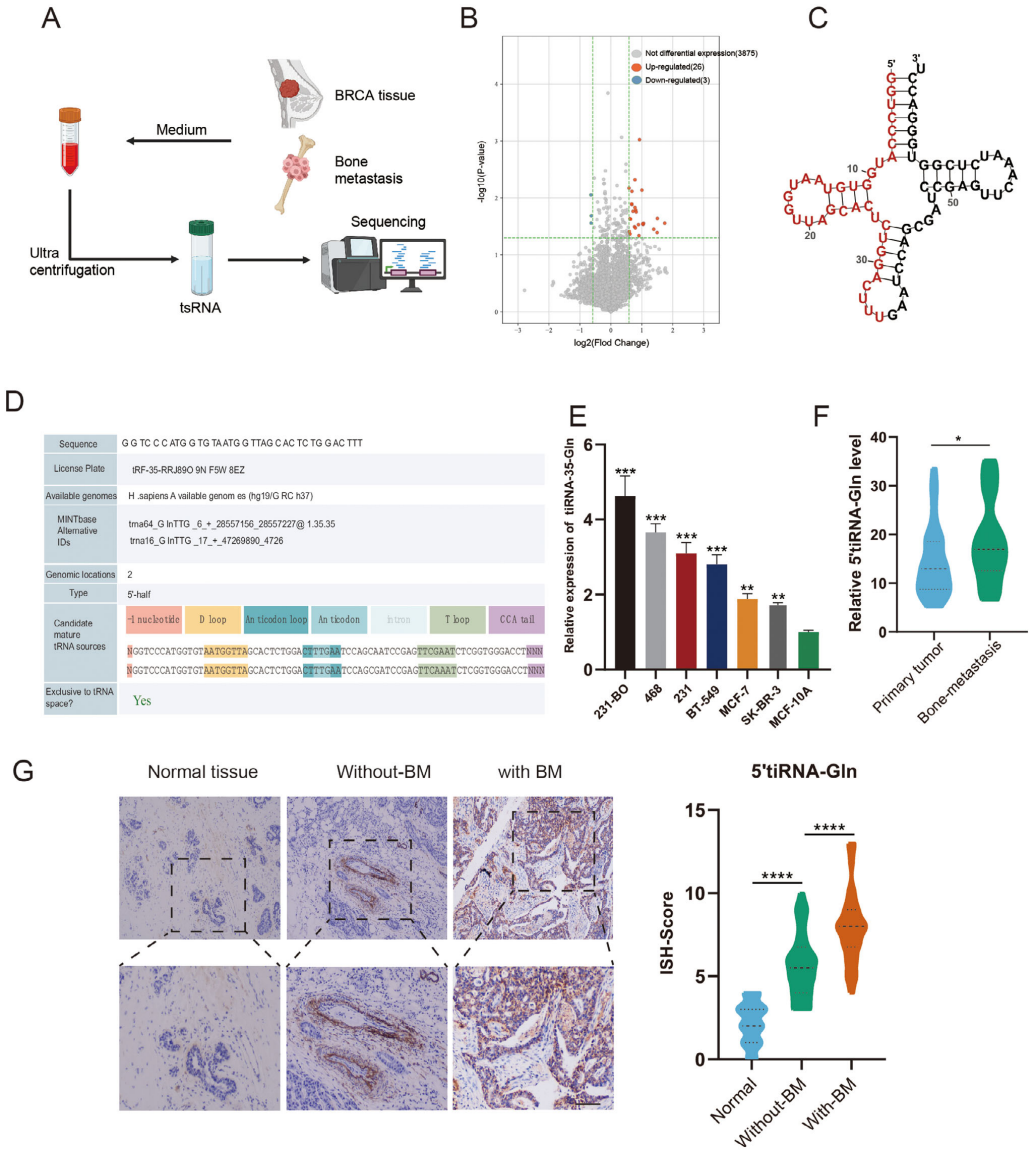
5’tiRNA‐Gln was related to BC bone metastasis. A: Flowchart of tsRNA sequencing. B: A total of 3914 tsRNA were identified by tsRNA sequencing, 26 were significantly regulated and 3 were downregulated in bone metastases compared to primary lesions; C: Secondary Structure of 5’tiRNA‐Gln (red labeled); D: 5’tiRNA‐Gln, derived from tRNAGln−TTG, belongs to the tiRNA‐5 fragment family; E: Comparison of 5’tiRNA‐Gln expression levels in different BC cell lines; F: The expression of 5’tiRNA‐Gln in BM tissues was significantly higher than that in paired primary tissues (n = 33); G: In situ hybridization (ISH) showed that 5’tiRNA‐Gln expression was significantly higher in breast cancer tissues with bone metastasis (n = 33) than in those without metastasis(n = 42). And 5’tiRNA‐Gln in BC tissues were significantly higher than that in paired adjacent normal tissues(n = 42). Scale bars: 100 µm. All data are presented as means±SD.

We next profiled 5’tiRNA‐Gln expression across a panel of human breast cancer cell lines with diverse metastatic potentials, alongside normal mammary epithelial cells (MCF‐10A). Quantitative analysis revealed that 5’tiRNA‐Gln expression was markedly enriched in the bone‐metastatic breast cancer cell line MDA‐MB‐231‐BO (231BO) (Figure 1E). To further validate this clinical relevance, we performed RT‐qPCR on an expanded cohort of 33 paired primary breast tumors and matched bone metastatic lesions, which robustly confirmed the marked upregulation of 5’tiRNA‐Gln in the metastatic bone lesions (Figure 1F). Seizing on its specific sequence, we designed a customized RNA in situ hybridization (ISH) probe targeting 5’tiRNA‐Gln and evaluated its spatial expression across paraffin‐embedded sections from 42 normal breast tissues, 42 non‐metastatic primary tumors, and 33 metastatic primary tumors. The ISH assays demonstrated that 5’tiRNA‐Gln expression was significantly higher in breast cancer tissues than in adjacent normal counterparts; importantly, its levels were further elevated in primary tumors that developed bone metastasis compared to non‐metastatic ones (Figure 1G). Taken together, these findings strongly suggest that 5’tiRNA‐Gln is clinically correlated with and potentially drives breast cancer bone metastasis.

### 2.2 5’tiRNA‐Gln Promotes BC Proliferation, Migration and Induces Osteoclast Differentiation

Subsequently, we explored the biological functions of 5’tiRNA‐Gln in BC cells. We first established stable 5’tiRNA‐Gln knockdown models in 231BO (high endogenous expression) and 231 cells, with knockdown efficiencies validated via RT‐qPCR (Figure S2A). Colony formation assays revealed that silencing 5’tiRNA‐Gln severely compromised the clonogenic capability of both cell lines (Figure 2A), whereas its ectopic overexpression markedly accelerated colony growth (Figure S2B). Concurrently, transwell migration and invasion assays demonstrated that 5’tiRNA‐Gln deficiency substantially impaired the migratory and invasive potentials of 231BO and 231 cells; conversely, forced overexpression of 5’tiRNA‐Gln in 231 cells robustly enhanced these metastatic traits (Figure S2C).

**FIGURE 2:**
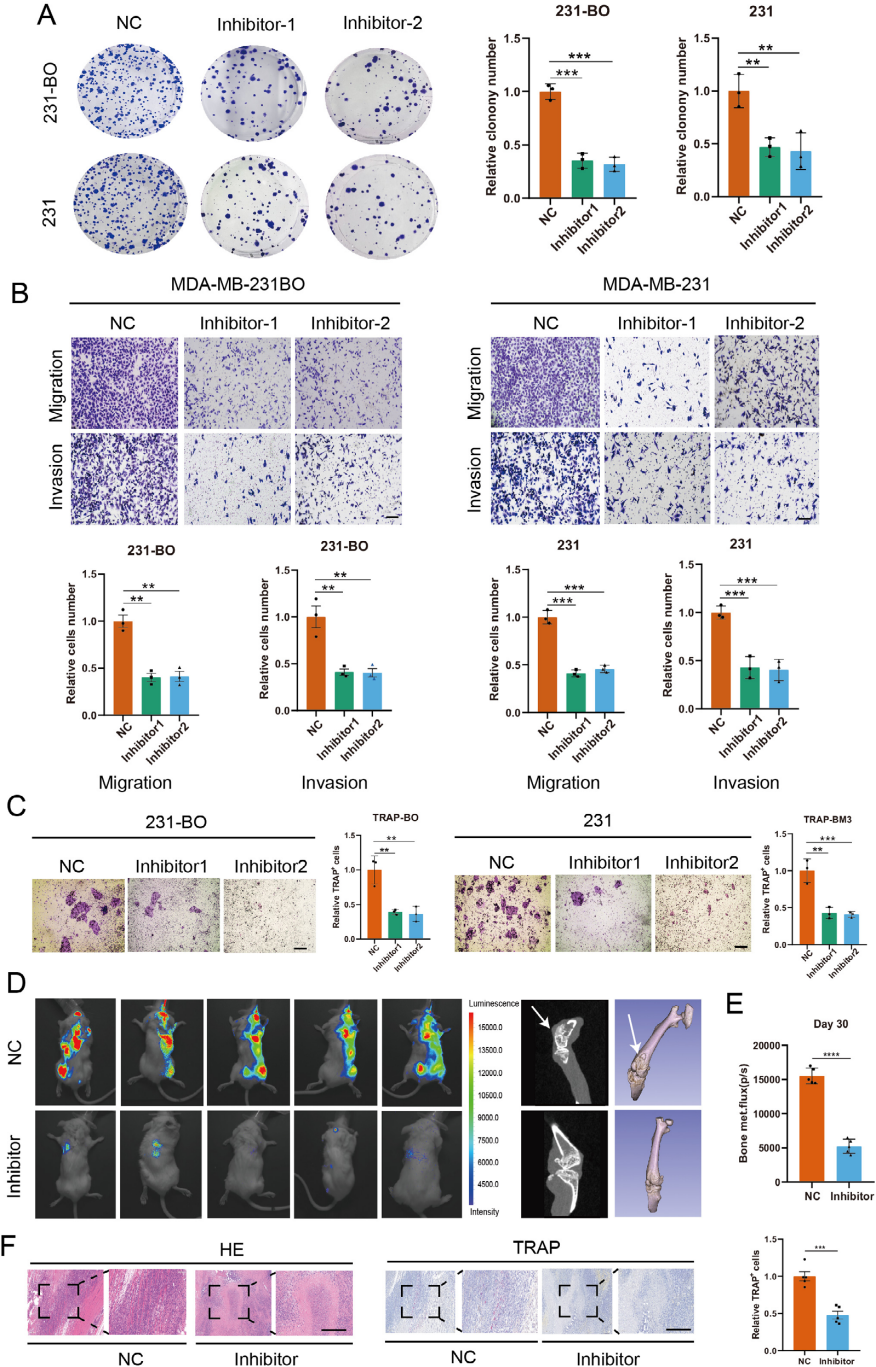
5’tiRNA‐Gln promotes the malignant phenotypes of BC cells. A, B: Colony formation and transwell assays demonstrated that suppression of 5’tiRNA‐Gln inhibited the proliferation rates, migration and invasion ability of 231BO and 231 cells; C: Representative staining images of mature TRAP+ osteoclasts after coculture with primary bone marrow cells and treatment of them with M‐CSF (30 ng/mL) and RANKL (30 ng/mL) for 6 days(n = 3). Scale bar, 200 µm; D: MDA‐MB‐231BO shCtrl and MDA‐MB‐231BO sh5’tiRNA‐Gln cells were transplanted into NCG mice via intracardiac injection. The growth of bone metastasis was monitored by In Vivo Imaging System (IVIS Bioimager); representative micro‐CT images were utilized to evaluate tumor‐induced bone osteolysis E: Quantitative bioluminescence analysis of tumor burden (fluorescence intensity); F: The TRAP staining was performed in tumor‐bearing bones from mice, together with H&E staining of the same field (n = 5). Scale bar, 100 µm.

Given that bidirectional communication between tumor cells and bone marrow‐derived stromal cells is a prerequisite for osteoclast differentiation and subsequent bone metastasis [20], we investigated whether 5’tiRNA‐Gln mediates these paracrine interactions. Tumor cell‐conditioned media (CM) was collected and co‐cultured with primary mouse bone marrow‐derived cells (BMMCs). Strikingly, CM derived from 5’tiRNA‐Gln‐deficient cells significantly blunted osteoclastic differentiation and maturation (Figure 2C), whereas CM from 5’tiRNA‐Gln‐overexpressing cells exerted the opposite, pro‐osteoclastogenic effect (Figure S2D). To rule out potential off‐target effects on the full‐length precursor and determine whether suppressing 5’tiRNA‐Gln affected its parental tRNA, we performed Northern blot analysis. The results confirmed that 5’tiRNA‐Gln inhibition did not induce alterations in tRNA‐Gln expression levels (Figure S2E).

To extend these findings in vivo, stable luciferase‐labeled 231BO cells bearing 5’tiRNA‐Gln knockdown were inoculated into NCG mice via intracardiac injection to establish a bone metastasis model. Bioluminescence imaging at day 30 post‐injection revealed that both the incidence and overall burden of bone metastatic lesions were dramatically lower in the 5’tiRNA‐Gln knockdown group than in the negative control (NC) group (Figure 2D,E). High‐resolution micro‐CT scanning and 3D structural reconstructions demonstrated severe, extensive osteolytic bone destruction in the distal femurs of the NC mice; in contrast, silencing 5’tiRNA‐Gln remarkably preserved trabecular and cortical bone integrity (Figure 2D). Consistent with the radiological evidence, histological evaluation via hematoxylin and eosin (H&E) and tartrate‐resistant acid phosphatase (TRAP) staining confirmed that 5’tiRNA‐Gln knockdown drastically suppressed intraosseous tumor growth and diminished the number of active, matrix‐resorbing osteoclasts at the tumor‐bone interface (Figure 2F). Collectively, these results robustly demonstrate that 5’tiRNA‐Gln acts as a potent oncogenic driver that promotes breast cancer proliferation, invasion, and osteoclastogenesis to facilitate bone metastasis.

### 2.3 5’tiRNA‐Gln Directly Interacted with the RRM Region of HNRNPC

To dissect the precise molecular mechanism by which 5’tiRNA‐Gln drives breast cancer bone metastasis, we performed an RNA pull‐down assay coupled with liquid chromatography‐mass spectrometry (LC‐MS) to identify its potential protein interacting partners. Biotinylated sense and antisense oligonucleotides corresponding to the 5’tiRNA‐Gln sequence were synthesized and incubated with 231BO cell lysates. Silver staining and Western blot analyses consistently revealed a distinct, specific protein band migrating at approximately 35 kDa in the sense pull‐down group compared to the antisense control (Figure 3A). Mass spectrometry analysis of the pulled‐down complexes identified a total of 177 candidate proteins (Figure 3B and Table S2). Among these, heterogeneous nuclear ribonucleoprotein C (HNRNPC) emerged as a top candidate due to its high peptide spectrum matches (PSMs) and prominent abundance corresponding to the 35 kDa band (Figure 3C). Data from the UniProt database annotated HNRNPC as a well‐established RNA‐binding protein featuring a conserved RNA recognition motif (RRM) spanning residues 16–87 (Figure S4A). The specific interaction between 5’tiRNA‐Gln and HNRNPC was further validated by Western blot of the pull‐down products (Figure 3D,E) and reciprocally confirmed by RNA immunoprecipitation (RIP)‐qPCR assays (Figure 3F,G). To delineate the binding region within HNRNPC, we constructed a series of FLAG‐tagged deletion mutants, including Δ1–87aa, Δ87–155aa, Δ162–306aa, and full‐length HNRNPC (Figure 3H). Domain mapping analyses demonstrated that the RRM domain (16–87aa) is indispensable for the interaction, as its deletion completely abolished the binding to 5’tiRNA‐Gln (Figure 3I). Consistently, RIP‐qPCR assays tracking these truncated constructs in 231BO cells verified that the interaction is strictly restricted to the RRM region (Figure 3K). To visualize this macromolecular interaction, AlphaFold‐based structural modeling was employed, predicting a stable 3D docking conformation between 5’tiRNA‐Gln and the HNRNPC RRM pocket (Figure 3J). Given that HNRNPC is predominantly a nuclear resident, we examined the spatial distribution of 5’tiRNA‐Gln via immunofluorescence (IF) combined with RNA fluorescence in situ hybridization (FISH). The results revealed that 5’tiRNA‐Gln is mainly localized in the nucleus and exhibits a striking spatial colocalization with HNRNPC, yielding robust Pearson's correlation coefficients (R) of 0.77 and 0.75, respectively (Figure 3L). Collectively, these results illustrated that 5’tiRNA‐Gln directly binds to the RRM region of HNRNPC. To further validate whether 5’tiRNA‐Gln exerts its oncogenic effects through HNRNPC, we utilized MDA‐MB‐231 cells, which exhibit relatively lower endogenous expression of these targets. Overexpression of 5’tiRNA‐Gln significantly promoted cell proliferation and enhanced migration (Figure S3A, B). Notably, silencing HNRNPC in 5’tiRNA‐Gln overexpressing cells effectively attenuated the enhanced migration and invasion (Figure S3A). Consistently, the promotional effect of 5’tiRNA‐Gln on cell proliferative vitality was also diminished following HNRNPC knockdown (Figure S3B). Collectively, these results demonstrate that the oncogenic function of 5’tiRNA‐Gln is dependent on the presence of HNRNPC.

**FIGURE 3:**
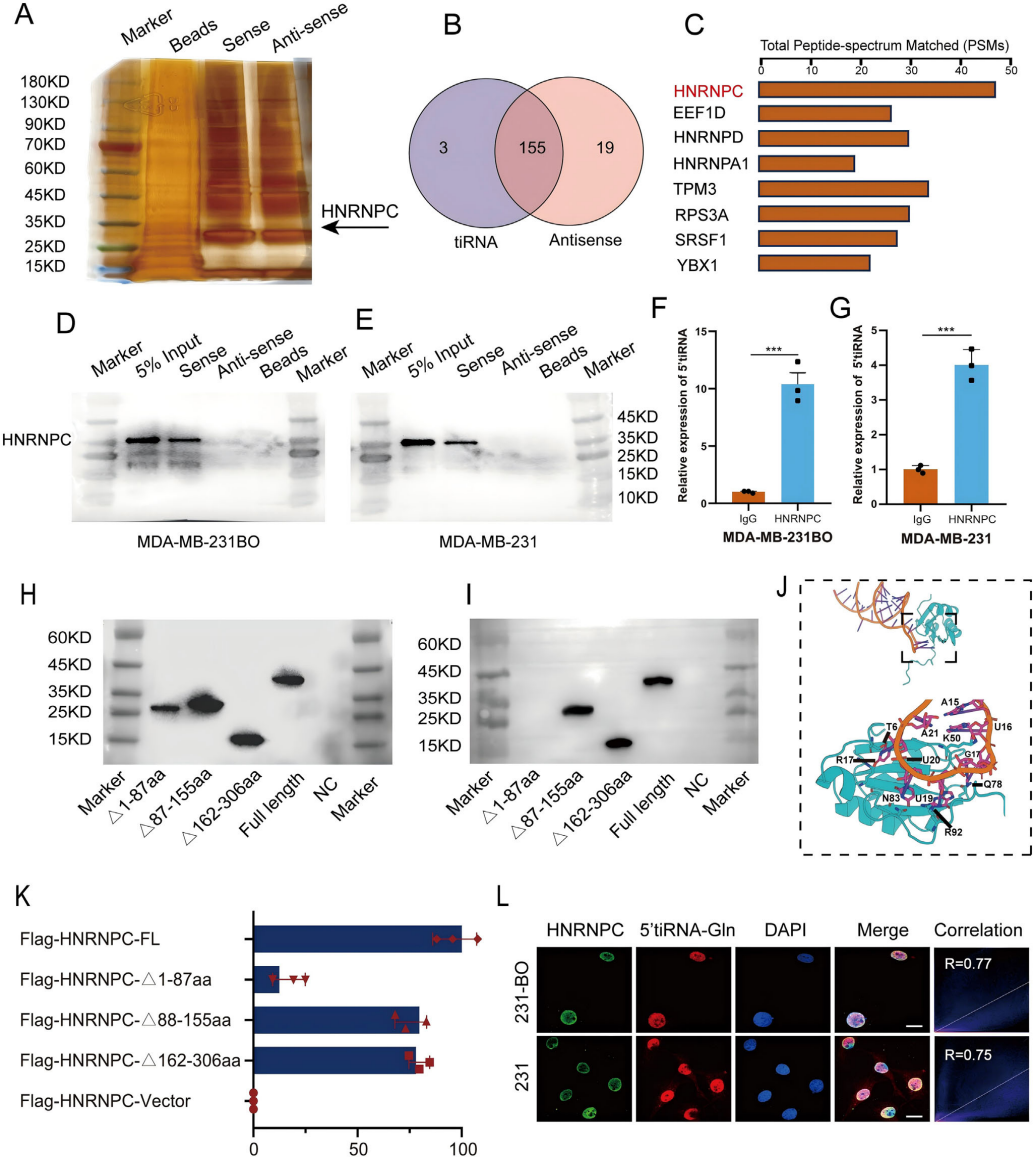
5’tiRNA‐Gln directly interacted with HNRNPC. A: Silver‐stained SDS‐PAGE gel images showed proteins immunoprecipitated by 5’tiRNA‐Gln and its antisense RNA in 231BO cells; B: Comparison of proteins enriched by 5’tiRNA‐Gln and its antisense control; C: Among the proteins enriched by 5’tiRNA‐Gln, HNRNPC exhibited the highest number of matched peptides and the greatest fold change; D, E: Western blot analysis of products from RNA pull‐down assays using 5’tiRNA‐Gln sense and antisense confirmed that 5’tiRNA‐Gln directly associated with HNRNPC in 231BO (D) and 231(E) cells (*Note*: Panels D and E were loaded on the same gel membrane and share the same middle marker lane); F, G: RIP followed by RT‐qPCR demonstrated the specific binding of 5’tiRNA‐Gln to HNRNPC in 231BO (F) and 231 (G) cells; H: Truncated HNRNPC plasmids were constructed based on the predicted functional domains, and the efficiency of plasmid expression was verified; I: RNA pull‐down and western blot assays revealed that 5’tiRNA‐Gln bound to the RRM domain of HNRNPC in 231BO cells; J: Predicted 3D structure of the HNRNPC–5’tiRNA‐Gln complex generated by AlphaFold3. HNRNPC is shown in blue, and 5’tiRNA‐Gln in orange; K: RIP‐qPCR demonstrated specific binding of 5’tiRNA‐Gln to RRM domain of HNRNPC in 231BO cells; L: Immunofluorescence analysis revealed a strong colocalization between HNRNPC (green) and 5’tiRNA‐Gln (red) in 231BO and 231 cells. Nuclei were counterstained with DAPI (blue).

### 2.4 HNRNPC Undergoes Liquid–Liquid Phase Separation

Given that 5’tiRNA‐Gln is moderately expressed in MDA‐MB‐231 and BT‐549 cells, we initially modulated its abundance via gain‐ and loss‐of‐function approaches to evaluate its impact on HNRNPC. Intriguingly, altering 5’tiRNA‐Gln expression levels affected neither the total protein/mRNA abundance nor the nucleocytoplasmic localization of HNRNPC (Figure 4A,B). This disconnected phenotype strongly indicated that 5’tiRNA‐Gln regulates HNRNPC through an unconventional post‐translational or biophysical mechanism rather than altering its expression. Heterogeneous nuclear ribonucleoproteins (hnRNPs) constitute a large family of RNA‐binding proteins that dictate cancer progression by modulating RNA splicing and transcription [21, 22]. Among them, HNRNPA2, HNRNPL and HNRNPK exhibit liquid‐liquid phase separation (LLPS), which contributes to tumor development [23, 24, 25]. PhaSePred database predicted that HNRNPC similarly possesses extensive intrinsically disordered regions (IDRs) with a remarkably high propensity for LLPS (Figure S4B).

**FIGURE 4:**
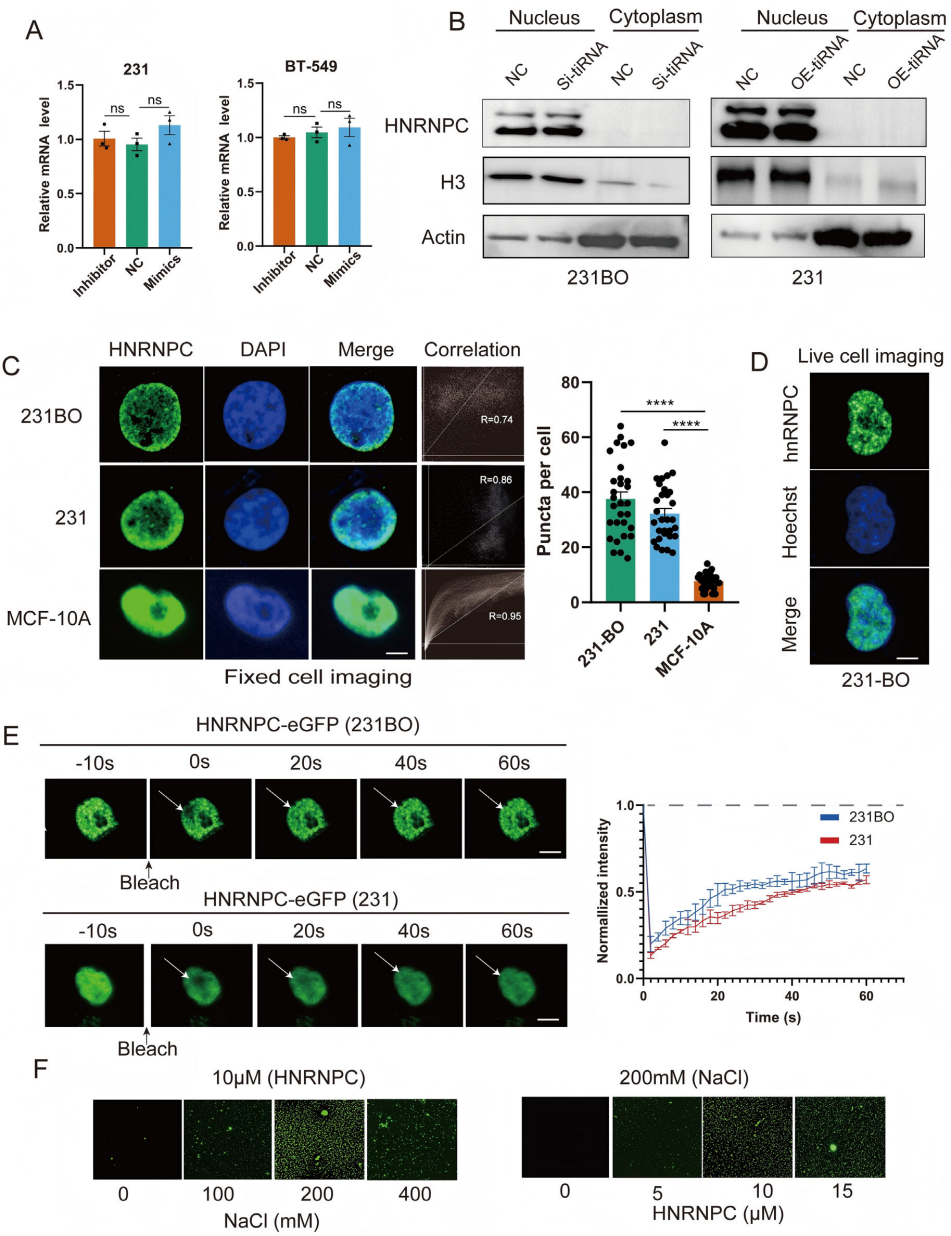
HNRNPC undergoes liquid–liquid phase separation. A, B: The mRNA(A) and protein (B) Expression levels of HNRNPC remained unchanged in MDA‐MB‐231 and BT‐549 cells transfected with 5’tiRNA‐Gln mimics or inhibitor; C: Immunofluorescent staining of HNRNPC in 231BO, 231 breast cancer cells and MCF‐10A normal breast cells. The right panel displays a plot illustrating the colocalization of HNRNPC‐GFP and DAPI and Pearson correlation coefficient analysis. Histogram revealed that quantitative analysis of HNRNPC puncta in three cell lines (n = 30 cells for each group); Scale bar, 5 µm; D: HNRNPC‐eGFP knock in in 231BO cells using the lentiviral transfection and representative images of HNRNPC in living cells. Scale bar, 5 µm; E: Representative images of fluorescence recovery and plot of fluorescence intensity of HNRNPC‐GFP droplets in 231BO cells after photobleaching over time. The blue curves represent the mean of the normalized fluorescence intensity in the photobleached region in distinct droplets (n = 3 independent experiments). Scale bar, 5 µm. Region of Interest (ROI) of 1 µm in diameter within the droplets was photobleached using a 488 nm laser at 40% laser power for a duration pulse of 1 s; F: Droplet formation of N‐terminally EGFP‐tagged HNRNPC. Protein concentration: 10 µM, Concentration of protein solution: 0 mM, 100 mM, 200 mM, 400 mM. Scale bar, 50 µm.

To test this hypothesis, we first performed immunofluorescence staining to examine endogenous HNRNPC organization. We observed that HNRNPC assembled into discrete, punctate condensates within the nuclei of breast cancer cells, whereas it remained diffusely dispersed throughout the nucleoplasm of normal breast epithelial cells (Figure 4C). To visualize this in real time, we transfected BC cells with a plasmid encoding EGFP‐tagged wild‐type HNRNPC (Figure S4C). Consistently, exogenous EGFP‐HNRNPC readily organized into spherical, distinct foci in live cells (Figure 4D). Disrupting hydrophobic interactions with the phase‐separation inhibitor 1,6‐hexanediol (1,6‐HD) significantly dissolved these nuclear HNRNPC condensates (Figure S4D). To assess the dynamic liquid‐like properties of these cellular EGFP‐HNRNPC foci, we performed fluorescence recovery after photobleaching (FRAP) assays. Upon laser photobleaching, the fluorescence signal within the bleached region rapidly recovered and reached a stable plateau within 1 min (Figure 4E), confirming that these nuclear HNRNPC foci possess high internal molecular mobility characteristic of liquid‐like condensates.

To definitively substantiate the intrinsic LLPS capacity of HNRNPC, we purified recombinant EGFP‐tagged wild‐type HNRNPC protein from 293T cells for in vitro reconstitution assays. HNRNPC spontaneously condensed into typical spherical droplets in a salt‐dependent manner; droplet formation initiated at 50 mM NaCl, peaked at approximately 200 mM, and progressively declined at higher ionic strengths (Figure 4F). Furthermore, HNRNPC phase separation exhibited strict concentration dependence, generating small, sparse droplets at low protein concentrations that shifted toward larger, coalesced macromolecular assemblies as the protein concentration increased (Figure 4F). Taken together, these in vitro and in vivo findings unequivocally demonstrate that HNRNPC undergoes bona fide liquid–liquid phase separation, uncovering a novel biophysical state of HNRNPC in breast cancer cells.

### 2.5 IDR2 Contribute to the LLPS Behavior of HNRNPC

To explore the definitive structural determinants driving HNRNPC phase separation, we analyzed its primary sequence using the IUPred2A algorithm [26]. HNRNPC was predicted to harbor two distinct intrinsically disordered regions (IDRs): a proline‐rich IDR1 (residues 139–190) and an acidic IDR2 (residues 221–306) (Figure 5A). The LLPS properties of proteins are mainly controlled by the IDR regions [27]. The molecular forces driving LLPS vary across different RNA‐binding proteins: such as glycine/proline‐rich motifs in hnRNPL/Pau [24, 28], or charged glutamic/aspartic acid clusters in DDX21/WDR3 [29, 30]. Since acidic mutation at IDR can change phase separation property of proteins [31, 32], we designed specific mutants based on the sequence features of HNRNPC. Specifically, we generated HNRNPC mutant in which all proline residues in the Pro‐enriched IDR1 were substituted with alanine. Additionally, we mutated IDR2 by replacing all negatively charged Glu and Asp residues with alanine (Figure 5B; Figure S4E). We generated HNRNPC expressing constructs and tagged them with EGFP, including HNRNPC with IDR1‐MUT (139‐190aa), HNRNPC with IDR2‐MUT (221‐306aa) (Figure 5B, Figure S4E). Consistent with wild‐type HNRNPC, IDR1‐MUT was still able to form spherical liquid‐like droplets, and FRAP assays indicated that its phase separation ability was preserved (Figure 5C,D). In contrast, IDR2‐MUT abolished droplet formation and prevented spontaneous fluorescence recovery (Figure 5C,D). Moreover, we generated the rescued HNRNPC mutant by fusing the IDR of hnRNPA1 (Mut‐IDR) that is known to drive condensate formation [33]. This replacement fully rescued phase separation ability by both droplet morphology and FRAP recovery (Figure 5C,D), confirming the critical role of IDR2, rather than IDR1 in HNRNPC condensation.

**FIGURE 5:**
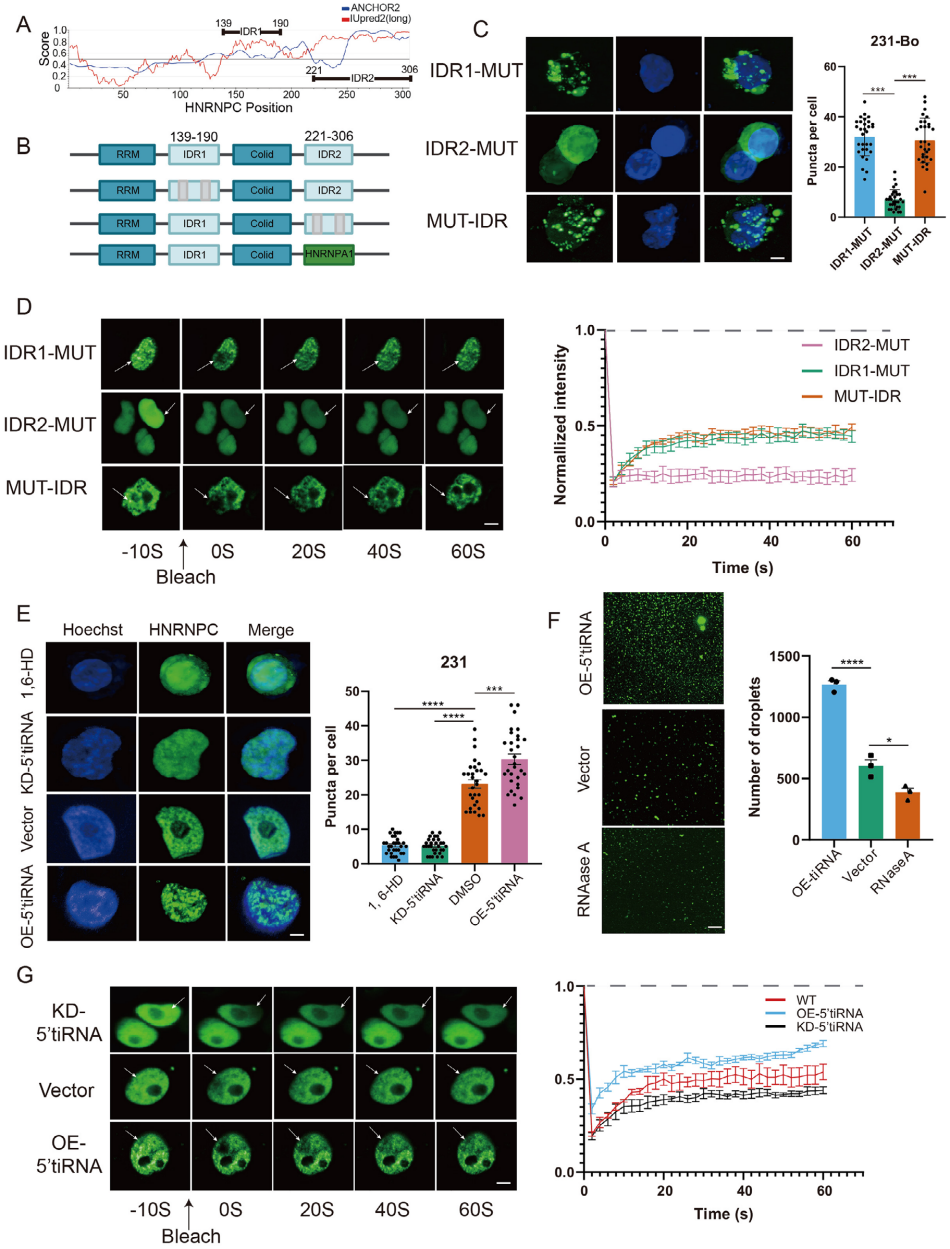
HNRNPC phase separation is dependent on its IDR2 domain. A: Prediction of intrinsic disorder tendency of HNRNPC by IUPred2A (https://iupred2a.elte.hu/). Scores above 0.5 indicate disorder; B: Schematic diagrams of wild‐type HNRNPC (WT) and its mutant constructs (MUT‐IDR1, MUT‐IDR2, and IDR‐MUT); C: Representative images of exogenously expressed HNRNPC‐GFP and each variant in 231BO cells. Scale bar, 5 µm. Statistical analysis of the LLPS puncta number of each variant is shown in the right panel (n = 30 cells for each group); D: Images and quantification of droplet formation by different mutants of HNRNPC after photobleaching; E: Representative images illustrating the alteration of HNRNPC puncta following 5’tiRNA‐Gln change in 231 cells. Scale bar, 5 µm. Statistical analysis of the LLPS puncta number of each variant is shown in the right panel (n = 30 cells for each group); F: Droplet formation of N‐terminally EGFP‐tagged HNRNPC. Scale bar, 50 µm; Region of Interest (ROI) of 1 µm in diameter within the droplets was photobleached using a 488 nm laser at 40% laser power for a duration pulse of 1 s; G: FRAP analysis showing the changes in fluorescence recovery of HNRNPC droplets after 5’tiRNA‐Gln knockdown or overexpression. Confocal images were taken at indicated time points relative to photo bleaching (0). Scale bar, 5 µm.

Since non‐coding RNAs frequently act as biophysical scaffolds that promote the LLPS of RNA‐binding proteins through RRM interactions [24, 34], we next investigated whether 5’tiRNA‐Gln directly modulates HNRNPC phase separation. In living 231 cells, endogenous HNRNPC assembled into discrete nuclear puncta, which coalesced into significantly larger, macroscopically prominent condensates upon 5’tiRNA‐Gln overexpression (Figure 5E). Conversely, 5’tiRNA‐Gln knockdown or treatment with 1,6‐hexanediol (1,6‐HD) drastically dissolved these nuclear structures (Figure 5E). To validate these cellular observations under cell‐free conditions, we purified EGFP‐HNRNPC protein from 293T cells subjected to prior 5’tiRNA‐Gln manipulation. Parallel to our cellular findings, 5’tiRNA‐Gln depletion severely disrupted in vitro HNRNPC droplet formation, whereas its enrichment drastically promoted droplet clustering (Figure 5F). Furthermore, live‐cell FRAP assays demonstrated that 5’tiRNA‐Gln overexpression substantially accelerated the fluorescence recovery of nuclear HNRNPC foci (Figure 5G). Collectively, these data demonstrate that 5’tiRNA‐Gln acts as a potent structural accelerator that optimizes and enhances the liquid‐liquid phase separation of HNRNPC in breast cancer cells.

### 2.6 HNRNPC Expression is Increased in Breast Cancer Bone Metastases and is Directly Correlated with Decreased Overall Survival in Breast Cancer Patients

To determine the clinical relevance of HNRNPC in breast cancer, we first analyzed its expression profiles utilizing the UALCAN database [35]. HNRNPC was found significantly upregulated in BC tissues compared to adjacent normal counterparts at both the mRNA and protein levels (Figure S5A,B). We next evaluated the GSE2034 dataset, which comprises 286 BC cases with documented organ‐specific metastatic annotations. Intriguingly, HNRNPC mRNA expression was significantly higher in primary tumors from patients who subsequently developed bone metastasis than in those with other metastatic patterns (Figure S5C). Consistently, HNRNPC baseline expression was highly enriched in the bone‐metastatic 231BO cell line compared to regular BC lines both at the mRNA and protein levels (Figure S5D,E). To robustly validate these findings, we performed immunohistochemical (IHC) staining on an independent tissue cohort from FUSCC, including 33 paired primary tumors and matched bone metastases, alongside 42 primary tumors from patients without bone metastasis (Table S9). The IHC analysis demonstrated that HNRNPC protein was markedly enriched in bone metastatic lesions relative to patient‐matched primary tumors; notably, primary lesions with bone‐metastatic tropism exhibited significantly higher HNRNPC levels than non‐metastatic ones (Figure S5F). Kaplan‐Meier survival analysis further revealed that elevated HNRNPC expression correlated with significantly worse overall survival across all major breast cancer molecular subtypes, including Luminal A, Luminal B, HER2+, and TNBC (Figure S5G). In 231BO cells, the attenuation of malignant behaviors—such as colony formation, migration and invasion—induced by HNRNPC knockdown was effectively rescued by re‐expressing HNRNPC (Figure S5H,I).

To further determine whether HNRNPC could serve as an independent prognostic indicator for patients with breast cancer, a multivariate Cox regression analysis was performed by adjusting for critical clinicopathological features. As demonstrated in the forest plot (Figure S7C), after adjusting for age, TNM stage, tumor grade, and Ki‐67 expression, high HNRNPC expression remained a robust and independent prognostic risk factor (HR = 2.845, 95% CI: 1.554–5.207, p < 0.001). These findings establish that HNRNPC expression holds significant predictive power for patient prognosis independent of conventional clinical staging and pathological metrics. Beyond the independent prognostic value of HNRNPC, we also evaluated the clinical diagnostic efficacy of 5’tiRNA‐Gln in predicting bone metastasis. Receiver operating characteristic (ROC) curve analysis revealed that expression levels of 5’tiRNA‐Gln possessed remarkable predictive power for identifying breast cancer bone metastasis, achieving an area under the curve (AUC) of 0.835(95% CI: 0.747‐0.924, N = 75; Figure S7B). Collectively, these findings establish that both 5’tiRNA‐Gln and HNRNPC hold substantial clinical translation value as promising diagnostic biomarkers and independent prognostic drivers for breast cancer bone metastasis.

These findings suggest that HNRNPC may play a critical role in the process of tumor bone metastasis. To test whether the oncogenic phenotypes driven by HNRNPC are fundamentally dependent on its biophysical phase separation, we conducted genetic rescue experiments using our newly engineered mutants. Since HNRNPC is an essential gene for cell viability and thus refractive to complete homozygous knockout, we achieved stable depletion via lentiviral short hairpin RNA (shRNA) transduction in 231BO cells. Functional colony‐formation and transwell assays showed that HNRNPC silencing profoundly suppressed BC cell growth, migration, and invasion (Figure 6A,C).

**FIGURE 6:**
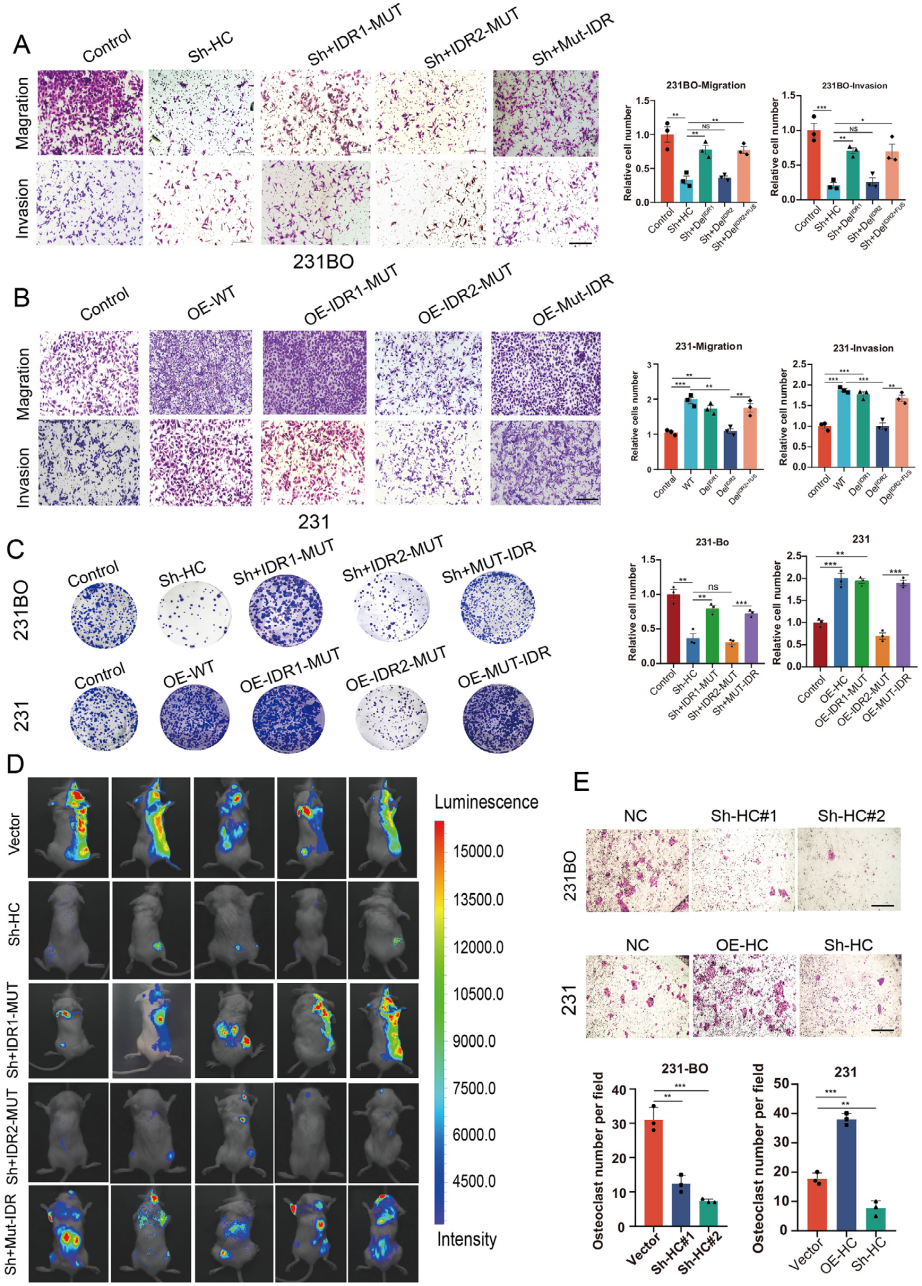
HNRNPC phase separation facilitates breast cancer progression. A: Transwell assays were performed to evaluate the migration and invasion abilities of HNRNPC‐knockdown 231BO cells rescued by overexpression of HNRNPC variants (MUT‐IDR1, MUT‐IDR2, and IDR‐MUT), compared with control cells; B: Transwell assay was performed to assess the migration and invasion ability of 231 cells with overexpressing WT, MUT‐IDR1, MUT‐IDR2, or IDR‐MUT HNRNPC variants vs. control group; C: Representative images of colony formation assays assessing the growth of BC cells following HNRNPC knockdown and subsequent overexpression of IDR1‐MUT, IDR2‐MUT, or MUT‐IDR variants in 231BO cells. In parallel, 231 cells were directly transfected with WT, IDR1‐MUT, IDR2‐MUT, or MUT‐IDR variants; D: HNRNPC‐knockdown 231BO cells overexpressing HNRNPC variants (MUT‐IDR1, MUT‐IDR2, and IDR‐MUT) were intracardiac injection into NCG mice. Tumors were harvested at day 30; E: The osteoclast differentiation ability of HNRNPC inhibition was assessed compared with control cells. Data are shown as means ± SD. *p* value was calculated by one‐way ANOVA test with multiple comparisons. n = 5 mice for each group.

Crucially, these aggressive phenotypes were fully restored by the re‐expression of IDR1‐MUT, but not by the phase‐separation‐deficient IDR2‐MUT (Figure 6A,C). Remarkably, the chimeric MUT‐IDR construct—wherein the defective IDR2 was replaced with the liquid‐forming IDR of hnRNPA1—successfully rescued the cell growth and metastatic traits (Figure 6A,C). Consistently, in xenograft tumor models, reintroduction of IDR1‐MUT, or MUT‐IDR successfully restored in vivo tumor expansion in HNRNPC‐depleted 231BO cells, whereas cells expressing the IDR2‐MUT variant remained severely growth‐attenuated (Figure 6D). Similarly, overexpressing wild‐type HNRNPC or IDR1‐MUT in 231 cells (which harbor low baseline HNRNPC) robustly accelerated cell growth and invasion, whereas IDR2‐MUT expression failed to induce these alterations (Figure 6B,C). Finally, we evaluated the role of HNRNPC in tumor‐stroma crosstalk. Conditioned media (CM) from HNRNPC‐knockdown BC cells significantly blunted the differentiation and maturation of mouse bone marrow cells into active osteoclasts, as resolved by TRAP staining, whereas CM from HNRNPC‐overexpressing cells exerted the opposite, stimulatory effect (Figure 6E). Together, these comprehensive clinical and functional data demonstrate that HNRNPC acts as a powerful promoter of breast cancer malignancy and bone microenvironment remodeling in a strictly phase‐separation‐dependent manner.

### 2.7 The 5’tiRNA‐Gln‐HNRNPC‐ IGF1R Axis Promotes Breast Cancer Progression

As an RNA‐binding protein, HNRNPC post‐transcriptionally coordinates gene expression landscapes by modulating transcript stability, alternative splicing, or translational efficiency [36, 37]. To discover the direct downstream targets of HNRNPC driving breast cancer bone metastasis, we executed a multi‐omics screening strategy integrating RNA‐seq, RIP‐seq, and eCLIP‐seq analyses in 231BO cells. RNA‐seq profiling identified 2,150 differentially expressed genes (DEGs) upon HNRNPC depletion, consisting of 470 significantly downregulated and 1,680 upregulated transcripts (Figure S6A). In addition, Gene Set Enrichment Analysis (GSEA) revealed that these transcriptomic alterations were highly enriched in the MEK/ERK signaling cascade (Figure 7A,B). Hyperactivation of the ERK pathway is well‐documented to not only trigger tumor cell invasion but also aggressively remodel the intraosseous microenvironment [38, 39]; it drives the secretion of matrix metalloproteinases (MMPs) to breach bone matrices and stimulates paracrine tumor expression of RANKL and IL‐8 to accelerate osteoclastogenesis [40, 41], which in turn co‐releases matrix‐bound growth factors (e.g., TGF‐β, VEGF) to propagate the osteolytic vicious cycle [42]. To determine how HNRNPC directly commands this pathway, we intersected the HNRNPC‐bound transcripts from our eCLIP‐seq and RIP‐seq datasets with the candidate DEGs from our RNA‐seq analysis. This stringent multi‐omic overlay converged on a refined core of 10 candidate genes directly docked and regulated by HNRNPC (Figure S6B). Among these, IGF1R (insulin‐like growth factor 1 receptor) stood out as the primary downstream effector, exhibiting both the most pronounced expression sensitivity and the highest‐confidence binding scores (Table S3). The eCLIP‐seq tracks resolved that HNRNPC directly targets the coding sequence (CDS) region of IGF1R mRNA (Figure 7D), recognizing a specific consensus motif (Figure 7C). To further validate this interaction, we performed a dual‐luciferase reporter assay by mutating the key nucleotides within the consensus motif (replacing C/A and G/T interchangeables, Figure 7E). Remarkably, the relative luciferase activity was significantly blunted following site‐directed mutagenesis (Figure 7F). Clinically, IGF1R is overexpressed in diverse malignant contexts and acts as a major upstream trigger of both PI3K/AKT and MEK/ERK cascades [43, 44, 45]. Analysis of the TCGA database revealed that HNRNPC expression positively correlated with IGF1R expression across all breast cancer molecular subtypes (Luminal A: R = 0.274; Luminal B: R = 0.316; HER2+: R = 0.272; TNBC: R = 0.165; Figure S6C). Our IHC validation confirmed that IGF1R protein levels were significantly elevated in clinical bone metastases relative to patient‐matched primary breast tumors (Figure S6D).

**FIGURE 7:**
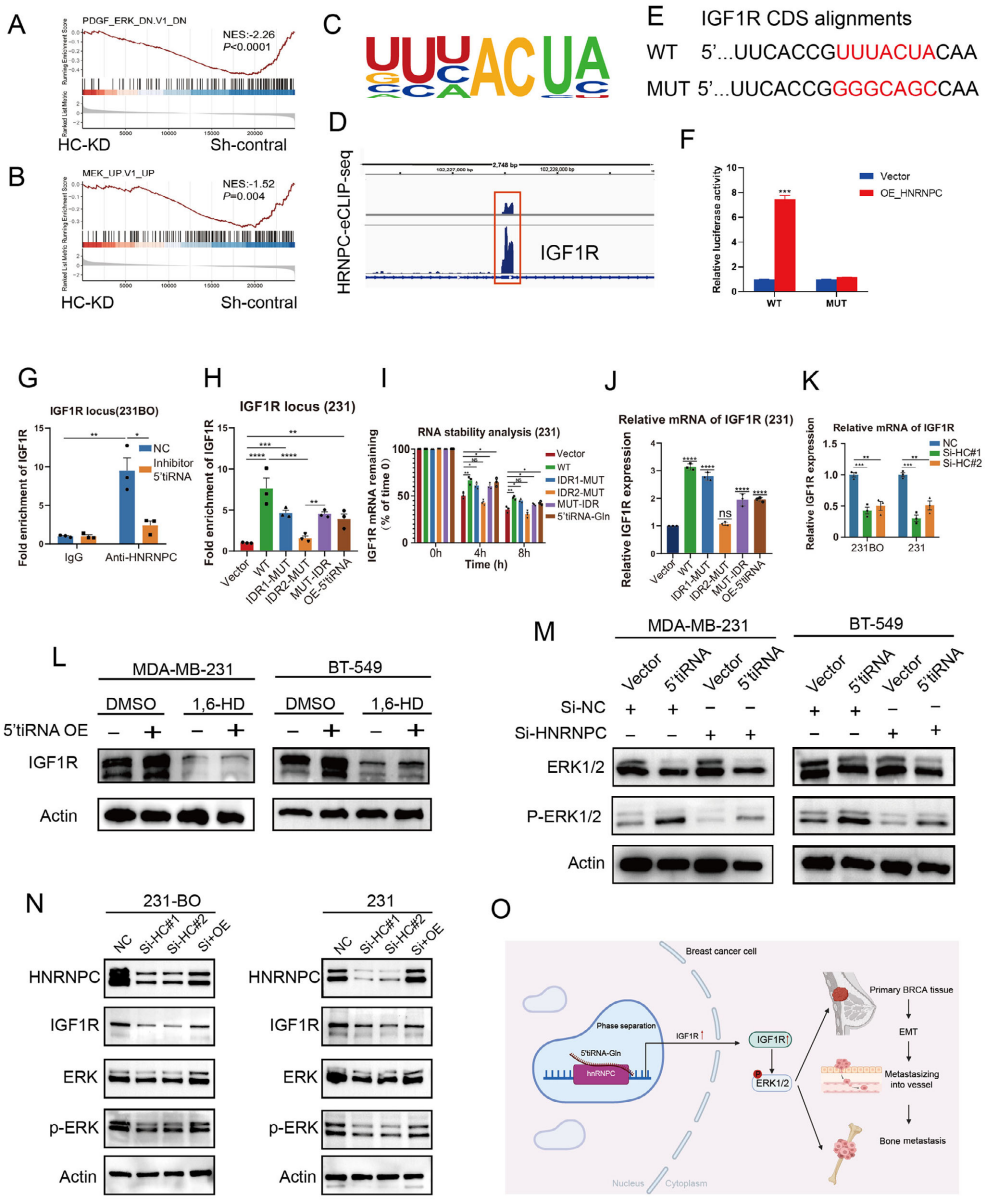
5’tiRNA‐Gln could facilitate HNRNPC binding to IGF1R and enhance the mRNA stability. A, B: Depletion of HNRNPC represses activation of MEK/ERK signaling; C: Sequence motif analysis revealed an UUUACUA consensus enriched in HNRNPC‐bound RNA fragments; D: Genome browser tracks (hg19) showing HNRNPC eCLIP peaks (lower track) relative to size‐matched (SM) input (upper track) on the IGF1R transcript; E: Schematic representation of wild‐type (WT) and mutant (MUT) binding motifs within the coding sequence (CDS) region of IGF1R mRNA. The predicted HNRNPC‐binding consensus sequence (red) was mutated to generate the MUT reporter construct; F: Relative luciferase activity in cells co‐transfected with WT or MUT IGF1R reporter plasmids together with empty vector or HNRNPC overexpression plasmid (OE_HNRNPC) (n = 3 independent experiments; mean ± SD; ***p < 0.001); G: RIP‐qPCR revealed that HNRNPC binds to the mRNA of IGF1R, and repression of 5’tiRNA‐Gln reduced this binding efficiency in 231BO cells; H: Overexpression of HNRNPC variants showed that WT and MUT‐IDR1 enhanced the binding of HNRNPC to IGF1R, whereas MUT‐IDR2 had no such effect. Fusion of HNRNPA1 to MUT‐IDR2 restored the binding‐promoting effect in 231 cells; I: Remaining IGF1R mRNA after actinomycin D treatment. Data represent mean ± SD; n = 3 independent experiments, one‐way ANOVA with multiple comparisons test; J–K: Relative IGF1R mRNA expression levels in 231BO and 231 cells following HNRNPC knockdown (K) or overexpression of HNRNPC WT and its mutants (J); L: Western blot of IGF1R upon overexpression of 5’tiRNA‐Gln or treated with 1,6‐HD in 231 and BT‐549 cells. 1,6‐HD: 1,6‐hexanediol; M: Western blot of ERK signaling pathway‐associated proteins in breast cancer cells upon overexpression of 5’tiRNA‐Gln or knockdown HNRNPC; N: Western blot analysis of IGF1R and p‐ERK expression following HNRNPC knockdown and subsequent re‐expression (rescue) in 231BO and 231 cells.; O: Schematic illustration depicting the molecular mechanism of the 5’tiRNA‐Gln/HNRNPC/IGF1R axis in mediating breast cancer bone metastasis via phase separation. Data are shown as means ± SD. *p* value was calculated by one‐way ANOVA test with multiple comparisons (E, F, G, H, I).

We next validated these eCLIP‐seq mappings using targeted RIP‐qPCR (Figure 7G). Intriguingly, acting as the upstream regulator of HNRNPC condensation, genetic depletion of 5’tiRNA‐Gln significantly weakened the physical binding affinity between HNRNPC and IGF1R mRNA in 231BO cells (Figure 7G). Reciprocally, in baseline‐low 231 cells, forced overexpression of 5’tiRNA‐Gln, HNRNPC‐WT, or the phase‐separation‐competent IDR1‐MUT—but notably not the phase‐separation‐deficient IDR2‐MUT—robustly enhanced HNRNPC enrichment at the IGF1R CDS region (Figure 7H). Mirroring this rescue trend, the MUT‐IDR fully restored HNRNPC‐binding capacity (Figure 7H). Although global splicing analyses indicated that the downstream MEK/ERK alterations were not primarily caused by aberrant alternative splicing of IGF1R, we hypothesized that HNRNPC phase separation instead dictates IGF1R transcript stability. Actinomycin D mRNA decay assays demonstrated that ectopic enrichment of HNRNPC‐WT, IDR1‐MUT, or 5’tiRNA‐Gln significantly prolonged the half‐life of IGF1R mRNA, whereas the LLPS‐defective IDR2‐MUT entirely failed to stabilize the transcript (Figure 7I). Again, the MUT‐IDR successfully rescued this transcript‐stabilizing function (Figure 7I). Consistent with the mRNA dynamics, RT‐qPCR verified that 5’tiRNA‐Gln, HNRNPC‐WT, IDR1‐MUT, and MUT‐IDR increased steady‐state IGF1R mRNA levels, whereas IDR2‐MUT exerted no effect (Figure 7J). At the translational level, overexpressing 5’tiRNA‐Gln bolstered IGF1R protein expression in both 231 and BT‐549 cells, a regulatory effect that was completely obliterated upon treatment with the phase‐separation inhibitor 1,6‐HD (Figure 7L). Conversely, HNRNPC knockdown plummeted IGF1R expression at both mRNA and protein levels, while re‐introducing HNRNPC fully salvaged both IGF1R abundance and downstream ERK phosphorylation (Figure 7K,N). To confirm that IGF1R mediates the oncogenic functions of HNRNPC, we overexpressed HNRNPC in MDA‐MB‐231 cells and subsequently knocked down IGF1R; this depletion effectively rescued HNRNPC‐driven enhancements in cell migration, invasion, and osteoclastogenesis (Figure S6E). Furthermore, pharmacological inhibition of the ERK pathway using HY‐12275 in 231BO cells markedly suppressed their migratory and invasive capacities (Figure S6F). Finally, functional Western blotting validated that 5’tiRNA‐Gln overexpression strongly hyperactivated downstream ERK signaling, whereas HNRNPC depletion thoroughly blocked this activation (Figure 7M). These findings were further clinically confirmed, as HNRNPC expression exhibited a significant positive correlation with p‐ERK levels in breast cancer bone metastasis lesions (R = 0.37, p = 0.035, Figure S7A). Collectively, these results substantiate that 5’tiRNA‐Gln licenses ERK pathway activation by nucleating HNRNPC phase separation, which structurally stabilizes IGF1R mRNA to perpetuate breast cancer bone metastasis (Figure 7O).

## 3 Discussion

Breast cancer remains a primary driver of cancer‐related mortality in women globally, second only to lung cancer [46]. Among distant metastatic cascades, the skeleton represents the most vulnerable and frequent site of recurrence [47]. Bone metastases trigger devastating skeletal‐related events (SREs), including intractable bone pain, pathological fractures, and spinal cord compression, which dramatically truncate patient life expectancy. Despite therapeutic leaps in primary tumor resections and adjuvant regimens, a substantial echelon of BC patients inevitably experiences localized bone relapse years later. Current standard‐of‐care frameworks—predominantly radiating metastatic foci or deploying osteoclast‐targeted agents such as bisphosphonates and denosumab (anti‐RANKL)—merely alleviate osteolytic lesions or delay SREs; unfortunately, they fail to arrest the upstream metastatic seed or tangibly extend overall surviva [9, 10]. Therefore, finding molecular targets to prevent and treat bone metastasis is urgently needed.

Emerging paradigms have highlighted tRNA‐derived small RNAs (tsRNAs), particularly tRNA fragments (tRNA fragments (tRFs) and tRNA halves (tiRNAs), as vital post‐transcriptional rheostats in malignant progression [49]. These non‐coding entities bypass classical microRNA‐like silencing to engage in multifaceted oncogenic networks. For instance, 3′tiRNA‐Leu directly docks onto the *RS28* transcript to hyperactivate its translational efficiency, while specific stress‐induced tRFs displace canonical RNA‐binding proteins (RBPs) like YBX1 from oncoprotein transcripts to selectively govern master regulators of metastasis [48]. Beyond these examples, tsRNAs buffer transcriptomic stability, translation fidelity, and ribosomal biogenesis to accommodate the metabolic demands of colonizing cells [49]. Our tsRNA sequencing of clinical samples unveiled a prominent enrichment of 5’tiRNA‐Gln within breast cancer bone metastases. Functional phenotyping validated that 5’tiRNA‐Gln serves as a bidirectional culprit: it autonomously fuels breast cancer cell migration and invasion while non‐cell‐autonomously remodeling the stromal niche by driving osteoclast maturation. Mechanistically, we discovered that 5’tiRNA‐Gln directly binds to the RNA recognition motif (RRM) of the heterogeneous nuclear ribonucleoprotein C (HNRNPC), placing this RBP at the center of the 5’tiRNA‐Gln driven metastatic program.

HNRNPC is a core component of the hnRNP complex and a ubiquitous orchestrator of nuclear RNA processing. While elevated HNRNPC expression routinely tracks with dismal survival in colon cancer [37], oral squamous cell carcinoma [50], and T‐cell leukemia [36], its exact role in breast cancer has remained historically controversial. Some studies initially framed HNRNPC as a putative tumor suppressor associated with favorable prognosis [51], whereas an expanding body of contemporary literature supports its role as an oncogenic driver [52, 53, 54]. By combining the GSE2034 dataset with our independent, clinically annotated FUSCC cohort, our study resolves this context‐dependent paradox. We demonstrated that HNRNPC expression scales progressively along the non‐metastatic‐to‐bone‐metastatic axis, peaking within the actual osseous lesions.

As an RNA‐binding protein (RBP), HNRNPC is well known for its functions in RNA splicing [55], stability [56], and translation [57]. Our study provides the first demonstration that HNRNPC undergoes liquid‐liquid phase separation (LLPS), a biophysical property undocumented for this specific hnRNP despite being well‐established in its familial relatives, HNRNPK, HNRNPL, and HNRNPA2 [23, 24, 25]. LLPS is critical for assembling membraneless organelles (MLOs) such as nucleoli, P‐bodies, and stress granules, which are involved in various physiological processes and pathological conditions [58]. Abnormal phase segregation is considered an important mechanism of gene regulation and can be involved in disease processes, including tumorigenesis [59]. Structurally, HNRNPC comprises an N‐terminal RRM, a central coiled‐coil domain, and two flanking intrinsically disordered regions (IDR1 and IDR2). The disordered region at the C‐terminus is long and enriched in charged amino acids Glu and ASP, which is critical for driving LLPS [60].

The C‐terminal IDR2 is highly enriched in negatively charged aspartic and glutamic acid clusters. FuzDrop forecasting (score: 0.996) and subsequent *in vitro/in vivo* phase assays confirmed that HNRNPC LLPS is completely dependent on this acidic IDR2. Intriguingly, unlike canonical LLPS drivers like FUS that rely heavily on hydrophobic β‐sheet stacking, HNRNPC condensation is primarily dictated by long‐range electrostatic forces. Most importantly, our rescue experiments proved that the oncogenic and metastatic capability of HNRNPC is completely lost upon IDR2 charge neutralization but can be fully restored by a heterologous phase‐separating IDR (MUT‐IDR). This indicates that macro‐molecular condensation provides the obligate structural architecture required to sustain its malignant phenotypes.

A particularly compelling facet of our study is the mechanism by which 5’tiRNA‐Gln dictates HNRNPC phase boundaries. While long non‐coding RNAs (e.g., *ZNF252P*) or structured circular RNAs (e.g., *circDNA2*) act as expansive molecular scaffolds that bridge multivalent RBP interactions to induce LLPS [34], 5’tiRNA‐Gln is comparatively short (35 nt). AlphaFold 3 modeling suggested that it docks into the HNRNPC RRM pocket via a localized hydrogen‐bonding network governed by its UUUACUA base motif, rendering multi‐valent scaffolding unlikely. Instead, we propose several non‐mutually exclusive biophysical mechanisms: Conformational Remodeling: The specific nucleotide composition of 5’tiRNA‐Gln engages the RRM to trigger an allosteric shift, fully exposing the C‐terminal IDR2 or the oligomerization interfaces and lowering the saturation concentration required for phase separation; Electrostatic Charge Screening: The highly charged phosphate backbone of the short tiRNA locally screens repulsive electrostatic forces among adjacent HNRNPC homodimers, shifting the phase boundary toward physiological protein concentrations; Viscosity and Local Ionic Shifts: Short RNA tracking alters the local dielectric and viscosity environment inside the nucleus, effectively nucleating RNP network structures into stable condensates.

Downstream of this phase‐separated compartment, we identified the insulin‐like growth factor 1 receptor (IGF1R) as the primary client transcript stabilized by the HNRNPC condensate. By integrating RNA‐seq, RIP‐seq, and eCLIP‐seq datasets, we revealed that HNRNPC directly docks onto the coding sequence (CDS) of IGF1R mRNA. Phase‐separated HNRNPC assemblies physically safeguard IGF1R transcripts from degradation, prolonging their half‐life and amplifying steady‐state IGF1R protein levels, which subsequently cascades into hyperactivated downstream MEK/ERK signaling. Given that the activated ERK pathway is a major instigator of the osteolytic vicious cycle—simultaneously augmenting tumor‐derived MMP invasion and paracrine RANKL/IL‐8 release [38, 39, 40, 41, 42]—the 5’tiRNA‐Gln/HNRNPC/IGF1R axis represents a vital target for therapeutic intervention.

Despite the comprehensive mechanistic insights provided by this study, several limitations should be acknowledged. First, the clinical validation of the 5'tiRNA‐Gln/HNRNPC axis across all breast cancer molecular subtypes remains a challenge. Due to the long latency period—often spanning years or even decades—between primary surgery and the onset of bone metastasis, paired longitudinal specimens are exceptionally rare. Furthermore, surgical intervention for bone metastatic lesions is typically reserved for patients experiencing severe skeletal‐related events (SREs), further limiting the availability of these tissues. While our current cohort demonstrates a strong correlation, future studies with larger, subtyped‐stratified biobanks are necessary to confirm if this axis serves as a universal biomarker across Luminal or HER2+ subtypes.

Second, while we utilized the MDA‐MB‐231‐derived BO cell line—which were rigorously established through multiple rounds of in vivo selection to exhibit superior bone‐homing potential, our functional assays were primarily conducted in a triple‐negative breast cancer (TNBC) background. Given that MDA‐MB‐231 and 4T1 are the most widely recognized and aggressive model for bone metastasis research [61, 62, 63], it provided a robust platform for our mechanistic discovery. However, the potential role and regulatory network of 5'tiRNA‐Gln in non‐TNBC subtypes remain to be fully elucidated in future investigations using diverse cell models.

Looking forward, dissecting the pathological role of RNP phase separation creates a paradigm shift for therapeutic design. Historically, small‐molecule drug discovery has focused almost exclusively on rigid, druggable pockets. In contrast, targeting biomolecular condensates offers an elegant blueprint to conquer historically “undruggable” disordered proteins [64, 65]. Small molecules can be screened to disrupt condensate material properties, modulate IDR conformational dynamics, or shift phase boundaries [66, 67]. Advanced platforms like DropScan and phase‐specific proteolysis‐targeting chimeras (PROTACs) offer precise tools to selectively clear aberrant condensates [68, 69]. Ultimately, developing therapeutics that selectively dismantle the 5’tiRNA‐Gln/HNRNPC phase separation—potentially in synergy with existing MEK/ERK inhibitors or standard chemotherapeutics like cisplatin—holds immense promise to shatter the osteolytic vicious cycle and transform survival outcomes for patients with advanced breast cancer.

## 4 Conclusion

In summary, our study reveals a novel mechanism driving breast cancer bone metastasis. We demonstrated that 5′tiRNA‐Gln is significantly upregulated in bone metastatic tissues and functions as a critical pro‐metastatic factor. Mechanistically, 5′tiRNA‐Gln physically interacts with the RNA‐binding protein HNRNPC, serving as a “molecular glue” to accelerate its LLPS. This enhanced LLPS behavior of HNRNPC subsequently stabilizes downstream IGF1R mRNA, thereby hyperactivating the oncogenic ERK signaling pathway to drive osteoclast differentiation and organ‐specific metastasis. These findings underscore the clinical significance of the 5’tiRNA‐Gln/HNRNPC axis and position it as a promising therapeutic target for mitigating breast cancer bone metastasis, warranting further exploration of its translational applications.

## 5 Materials and Methods

### 5.1 Reagents, Antibodies, and Plasmids

Cell lines:293T, Hela, MDA‐MB‐231, BT549, MCF7, SkBr3 were acquired from the American Type Culture Collection (ATCC). These cells were cultured in complete Dulbecco's modified Eagle's medium (DMEM; Gibco) supplemented with 10% FBS (Gibco), 1% Penicillin–streptomycin–amphotericin B (NCM biotech) under 5% CO2 at 37°C. MDA‐MB‐231BO (bone metastatic derivatives) were generously provided by Gong Yang's laboratory at Fudan University Shanghai Cancer Center [70]. MCF‐10A cells were cultured in MCF‐10A Cell Complete Medium (DMEM/F12+5% HS+20 ng/mL EGF+0.5 µg/mL Hydrocortisone+10 µg/mL Insulin+1% NEAA+1% P/S, Procell, Wuhan, China).

Actinomycin D (Act.D), and Dimethyl sulfoxide (DMSO) were purchased from MedChemExpress (MCE, Shanghai, China). 1, 6HD (240117) and PEG8000 (PHR2894) were purchased from Merck (Darmstadt, Germany). RNA‐pull down kit was purchased from Thermo Fisher Scientific (21115). D‐Luciferin potassium salt, Puromycin Dihydrochloride were purchased from Beyotime Biotechnology (Shanghai, China).

Recombinant Mouse CSF‐1/M‐CSF Protein (RP01216) was purchased from ABclonal. RANKL Protein (310‐08‐100UG) was purchased from Thermo Fisher. The antibodies against Flag (8146), Erk1/2 (4695), p‐Erk1/2(4370) were purchased from Cell Signaling Technology (CST). Other antibodies used included HNRNPC (Proteintech, 11760‐1‐AP), IGF1R (Proteintech, 20254‐1‐AP), β‐Actin (Proteintech, 20536‐1‐AP), HRP‐conjugated affiniPure goat antirabbit IgG (Proteintech, SA00001‐2). The details of all antibodies used in this study are provided in Table S4.

Cellular models of tiRNA‐Gln overexpression and inhibition, along with their respective controls, were established by transfecting cells with tiRNA‐Gln mimics, inhibitors, or corresponding negative control (NC) oligonucleotides. All oligonucleotides were designed and synthesized by RiboBio (Guangzhou, China). The sgRNA, PCDH, and shRNA plasmids, along with the packaging plasmids, psPAX2 and pMD2.G, were transfected into HEK293T cells, respectively. The lentivirus was collected 24–48 h after transfection and then used for infection. pcDNA3.1 plasmids and siRNAs were transfected into cells following the corresponding protocols. The sequences of 5’tiRNA‐Gln mimics and Inhibitors are listed in Table S7. The shRNA obtained from GenePharma (Shanghai, China) sequences are listed in Table S8.

### 5.2 RNA Isolation and Quantitative Real‐Time PCR

Total RNA was isolated using TRIzol reagent (Invitrogen) following the manufacturer's protocol. Complementary DNA (cDNA) was synthesized from the extracted RNA with the HiScript II Q RT SuperMix for qPCR kit (Vazyme). Quantitative real‐time PCR was then carried out using SYBR qPCR Master Mix (Vazyme) on a CFX96 Real‐Time PCR Detection System (Bio‐Rad). The primer sequences used for RT‐qPCR are listed in Supplementary Table S5.

### 5.3 Western Blot

Cell lysates were prepared in RIPA buffer (beyotime) with 1X cocktail (MCE), and protein concentrations were quantified using a Pierce BCA protein assay kit. An equal amount of protein (20–40 µg) was used in western blot analysis.

### 5.4 Immunofluorescence (IF) Assay

Cancer cells were seeded onto glass coverslips placed in 24‐well plates at a density of 1.5 × 10^4^ cells per well and cultured for 24 h. Cells were then fixed with 4% paraformaldehyde for 20 min at room temperature, followed by three washes with phosphate‐buffered saline (PBS). Permeabilization was performed using 0.1% Triton X‐100 (200 µL per well) for 10 min. After washing, cells were blocked with 5% bovine serum albumin (BSA) for 1 h at room temperature to prevent nonspecific binding. Subsequently, cells were incubated with an anti‐HNRNPC primary antibody (1:100 dilution) overnight at 4 °C. After primary antibody incubation, cells were washed and incubated for 1 h at room temperature with a fluorescein isothiocyanate (FITC)‐conjugated goat anti‐rabbit IgG secondary antibody (SA00003‐2, Proteintech). Finally, cell nuclei were counterstained with DAPI for 10 min.

### 5.5 Osteoclast Differentiation

Female C57BL/6 mice aged 4 to 6 weeks were used for this experiment. The mouse was euthanized and then immersed in 75% ethanol for 3–5 min for surface disinfection. The skin of both hind limbs was incised using ophthalmic scissors. A sterile 1 mL syringe was filled with 1 mL of HBSS (Hank's Balanced Salt Solution) to flush the bone marrow cavity to harvest bone marrow cells. Each bone was flushed 2–3 times. The bone marrow suspension was filtered through a 200 µm sterile cell strainer to remove debris. Then the filtered cells were resuspended in 1 mL ACK lysis buffer (NCM biotech, C100C3) to lyse red blood cells, incubated at room temperature for 3 min, followed by the addition of 9 mL HBSS. The sample was centrifuged again at 1350 rpm for 10 min, and the supernatant was discarded. Viable cells were counted and resuspended in α‐MEM complete medium (90% α‐MEM + 10% FBS+1% Penicillin–streptomycin–amphotericin B). Cells were seeded in 48‐well plates at a density of 50,000 cells per well in 1 mL complete medium. 50 ng/mL M‐CSF (ABclonal, Wuhan, China, RP01216) was added to the medium to induce macrophage attachment. Tumor cells were cultured in serum‐free DMEM for 24 h. The conditioned medium was collected, centrifuged, and filtered through a 0.45 µm syringe filter to remove debris and obtain tumor cell‐conditioned medium. After primary bone marrow cells adhered to the culture plate, the medium in 48‐well plates was removed. The conditioned medium and α‐MEM complete medium were mixed at a ratio of 1:10 and added to each well (1 mL per well). Recombinant RANKL (50 ng/mL) and M‐CSF (50 ng/mL) were supplemented into the medium to promote osteoclast differentiation. Cell morphology and status were monitored daily. On day 6, after multinucleated cells were observed under a microscope, the cells were washed with PBS, fixed with paraformaldehyde, and subjected to TRAP (tartrate‐resistant acid phosphatase) staining (G1050‐50T, Servicebio, Wuhan, China). Stained cells were observed and counted under a microscope.

### 5.6 RNA‐Protein Pull‐Down Assay

Biotin‐labeled tiRNA‐Gln sense and antisense sequences were obtained from Genescript (Nanjing, China). RNA pull‐down assays were conducted using a Pierce Magnetic RNA–Protein Pull‐Down Kit (no. 20,164, Thermo Fisher, MA, USA) according to the manufacturer's instructions. Biotin‐labeled RNA–protein complexes were lysed in 20 µL of RIPA buffer, followed by the addition of 5 µL of 5×loading buffer. The samples were then boiled for 10 min and subjected to SDS‐PAGE. After electrophoresis, the gel was collected and subjected to silver staining. Differential protein bands between the sense and antisense groups were excised and submitted for mass spectrometry analysis (Applied Protein Technology, Shanghai, China). Peptides were reconstituted in 0.1% formic acid and analyzed using liquid chromatography coupled with tandem mass spectrometry (LC‐MS/MS) on Thermo Fisher Orbitrap Fusion. The MS data were analyzed using MaxQuant software (version 1.6.14).

### 5.7 RNA immunoprecipitation (RIP)

EZ‐Magna RIP RNA Binding Protein Immunoprecipitation Kit (17–700, Millipore) was used to perform RIP assays following the protocol. Anti‐HNRNPC antibody and normal mouse IgG were used to immunoprecipitated with target RNA or as a negative control. Finally, RNA was subjected to qRT‐PCR analysis to detect the enrichment between indicated RNA binding to HNRNPC. Primers and probes for Rip‐qPCR assays were listed in Table S6.

### 5.8 RNA Stability Assay

Cells were treated with 5 µg/mL actinomycin D to block transcription. Cells were harvested at indicated time points and total RNA was extracted and used for qRT‐PCR. The relative abundance of each transcript was normalized to the level at time 0.

### 5.9 Animal Study

Female NOD scid gamma (NSG) immunodeficient mice (4–6 weeks old) were purchased from Sinogene (Beijing, China). Mice were randomly assigned to experimental groups prior to treatment. Mice were anesthetized with isoflurane and secured in a supine position. The left ventricle was located using a small animal ultrasound imaging system. A total of 1 × 10^6^ tiRNA‐Gln knockdown or control cells suspended in 100 µL PBS were injected into the left ventricle using an insulin syringe. 4 weeks after the injection, the mice were sacrificed to terminate the experiments. For bioluminescence imaging, potassium D‐luciferin solution was intraperitoneally injected at a dose of 10 µL per gram of body weight. After 10 min, vivo imaging was performed using a live animal imaging system. Tumors from both groups were dissected, fixed in formalin, embedded in paraffin, and subsequently subjected to hematoxylin and eosin (H&E) staining, and tartrate‐resistant acid phosphatase (TRAP) staining. All animal procedures were approved by the Ethics Committee for Animal Experiments of Fudan University, Shanghai Cancer Center.

### 5.10 Cell Migration and Invasion Assays

For colony formation assays, after cell counting, the cells were seeded into 6‐well plates (1000 cells per well) and then cultured for 2 weeks. The colonies were fixed with 4% Paraformaldehyde and stained with 0.2% crystal violet. Colonies were counted using the ImageJ software. For cell invasion assays, Matrigel (No. 356231, Corning) was diluted at a ratio of 1:8 with serum‐free medium, and 60 µL of the diluted Matrigel was added to the upper compartment of the Transwell chamber. 5–8 × 10^4^ cells were resuspended in a serum‐free medium and then plated in the upper chamber (#353097, Falcon), and a medium containing 20% FBS was added to the lower chamber. After incubation for 24 h, the cells in the upper chamber were fixed with 4% Paraformaldehyde and stained with 0.2% crystal violet. The migration assay was performed similarly to the invasion assay, with the exception that no Matrigel was applied to the upper chamber.

### 5.11 Cellular Phase Separation Assay

Cells expressing HNRNPC‐EGFP or HNRNPC‐Mut‐EGFP (IRDs mut) were seeded onto confocal dishes. After adherence, the cells were infected with 5’tiRNA‐Gln OE or vectors and finally analyzed by confocal microscopy.

### 5.12 Fluorescence Recovery after Photobleaching (FRAP) Assay

FRAP was performed using a Nikon NSPARC confocal microscope. Cells expressing green fluorescent signals were identified under a 100× objective. A bright fluorescent spot was selected and marked according to the program settings, followed by photobleaching with a high‐intensity laser. Specifically, a defined circular Region of Interest (ROI) of 1 µm in diameter within the droplets was photobleached using a 488 nm laser at 40% laser power for a pulse duration of 1 s. The moment of photobleaching was defined as 0 s. Subsequently, fluorescence recovery was recorded at 1‐second intervals for a total duration of 60 s, and the recovery curve was plotted based on the relative fluorescence intensity values over time.

### 5.13 In Vitro Phase Separation Assay

Recombinant protein was purified using Anti‐DYKDDDDK Magnetic Agarose Beads (SM009100, Smart‐lifesciences, Changzhou, China) according to the manufacturer's instructions. Purified protein was diluted to the indicated concentrations in storage buffer and mixed with 10% (w/v) PEG8000 (PHR2894‐1G, Sigma, USA). The mixtures were transferred into a confocal imaging dish, and droplet formation was examined using a confocal microscope.

### 5.14 Micro‐CT Scan

The harvested bone samples were analyzed using a high‐resolution micro‐CT system (Quantum GX2, PerkinElmer) equipped with a high‐sensitivity CdTe semiconductor detector. To minimize beam hardening, scans were performed at a tube voltage of 60 kV and a tube current within the 20–200 µA range, employing a 0.5 mm aluminum filter. Images were acquired using a 360° continuous rotation protocol, resulting in a reconstructed isotropic voxel size of 4.5 µm. The raw data were subsequently processed for 3D reconstruction and volumetric visualization using 3D Slicer software (version 5.10.0).

### 5.15 Northern Blot

Northern blotting was performed to detect the expression of tsRNA/tRNA and U6. Briefly, 10 µg of total RNA from each sample was denatured at 65°C for 10 min followed by instant chilling, then separated on a 15% denaturing urea‐polyacrylamide gel by electrophoresis at 150 V for 2 h. RNA was subsequently transferred onto a positively charged nylon membrane using a semi‐dry transfer system at 15 V for 30 min. Following transfer, the RNA was immobilized on the membrane by UV crosslinking at 120 mJ/cm^2^.The membrane was then cut into separate sections for different probes. Each membrane section was prehybridized in hybridization buffer for 2 h at 55°C, followed by hybridization with 300 pmol of the corresponding biotin‐labeled DNA antisense probe at 42°C overnight. The membranes were washed according to the manufacturer's instructions, and hybridization signals were detected using a chemiluminescent biotin detection system and imaged with a ChemiDoc XRS+ imaging system (Bio‐Rad).

### 5.16 Statistical Analysis

All statistical analyses were performed using R software (version 4.3.2) and GraphPad Prism (version 8.3). Data are presented as mean ± standard deviation (SD). Student's t‐test (two‐tailed, unpaired) was applied for comparisons between two groups. Continuous data were compared among multiple groups using one‐way analysis of variances (ANOVAs). The 2^−ΔΔCt^ method was used to analyze the results of qRT‐PCR analyses. Survival analyses were conducted using the Kaplan–Meier method, and statistical differences were assessed by the log‐rank test. Correlations between variables were evaluated using Pearson's correlation coefficient, depending on data distribution. A two‐sided p‐value < 0.05 was considered statistically significant. *p < 0.05; **p < 0.01; ***p < 0.001, ***p < 0.0001.

### 5.17 Patient Cohort

Clinical breast cancer tissues and matched bone metastasis (BM) samples were obtained from the FUSCC cohort. To evaluate the clinical relevance of 5'tiRNA‐Gln and HNRNPC, a comprehensive retrospective analysis was performed on patient baseline and clinicopathological characteristics. Molecular profiles of the primary tumors, including the expression status of estrogen receptor (ER), progesterone receptor (PR), and human epidermal growth factor receptor 2 (HER2), were clinically evaluated and documented. Concurrently, the molecular expressions of key biomarkers in the corresponding bone metastatic lesions were assessed via immunohistochemistry or in situ hybridization. The detailed clinicopathological characteristics and treatment histories are summarized in Supplementary Table S9. This study was approved by the Institutional Review Board, and informed consent was obtained from all participants.

## Author Contributions

W.J.Y., P.Z. and B.N.W. designed the study; D.S.L., Y.W., and P.Z. drafted and revised the paper; X.Z.F., X.S.L., J.L.F., and M.C. provided acquisition, analysis and interpretation of data, and statistical analysis; Z.X.W. and W.Y. provided technical and material support. All authors read and approved the final paper.

## Funding

This work was supported by the National Natural Science Foundation of People's Republic of China (8257114622).

## Conflicts of Interest

The authors declare no conflicts interest.

## Supporting information




**Supporting File 1**: advs77974‐sup‐0001‐SuppMat.docx.

**Supporting File 2**: advs77974‐sup‐0002‐SuppMat.zip.

**Supporting File 3**: advs77974‐sup‐0003‐SuppMat.pptx.

## Data Availability

The data that support the findings of this study are available from the corresponding author upon reasonable request.
