# Supplementary material for: Transfer RNA‐Derived Small RNA‐5’tiRNAGln Drives HNRNPC Phase Separation to Promote Breast Cancer Bone Metastasis by Regulating IGF1R
Source: Adv Sci (Weinh). 2026 Sep 29:e77974. Online ahead of print. doi: 10.1002/advs.77974 (PMC13624320; doi:10.1002/advs.77974)
Supplement: Supplementary file 1 [file ADVS-9999-e77974-s001.docx]

**Supplemental figures**

**Figure S1. Overview of tsRNAs identified from three paired tsRNA-sequencing samples.** (A) Among the identified tsRNAs, the most abundant subclass was tRF-5 (1,595 species), followed by 5′tiRNAs (778 species) and tRF-3 (709 species); (B) The majority of tsRNAs were derived from tRNA^Cys-GCA (N = 207), which carries cysteine with the anticodon GCA; (C) Length distribution of tsRNAs. tsRNAs with a length of 20 nucleotides were the most abundant, totaling 686 molecules.

**Figure S2. Validation of 5′tiRNA-Gln knockdown and overexpression efficiency and the functional effects of its overexpression.** (A) Validation of 5′tiRNA-Gln knockdown efficiency in 231BO and 231 cells and overexpression efficiency in 231 cells; (B) Colony formation assays demonstrated that overexpression of 5’tiRNA-Gln increased the proliferation rates of 231 cells; (C) 5’tiRNA-Gln overexpression promote cell migration and invasion in 231 cells; (D) Representative staining images of mature TRAP + osteoclasts after coculture with primary bone marrow cells and treatment of them with M-CSF (30 ng/mL) and RANKL (30 ng/mL) for 5 days. (n = 3) Scale bar, 200 μm; (E)Validation of 5’tiRNA-Gln knockdown by Northern blot. MDA-MB-231BO cells were treated with NC or 5’tiRNA-Gln inhibitor. Left: PAGE gel tracking dyes and estimated RNA marker sizes. Right: Northern blot detection of mature tRNA-Gln, 5’tiRNA-Gln (~35 nt), and U6 snRNA (loading control).

**Figure S3:HNRNPC is essential for 5’tiRNA-Gln-mediated promotion of breast cancer malignancy.** Representative images (left) and quantification (right) of transwell migration invasion and cell proliferation assays in MDA-MB-231 cells. Overexpression of 5’tiRNA-Gln (OE-5’tiRNA-Gln) significantly enhanced cellular migration, invasion and cell proliferation (B), simultaneous knockdown of HNRNPC (OE+siHC) effectively abrogated these pro-metastatic effects. SiHNC:Si-HNRNPC. Data are presented as mean±S.D. from three independent experiments. *p < 0.05; **p < 0.01; ***p < 0.001, ***p < 0.0001.

**Figure S4. Protein structure of HNRNPC and validation of its liquid–liquid phase separation potential.** (A) Domain organization of HNRNPC predicted from the UniProt database, including RNA recognition motif (RRM), coiled-coil, and acidic residue regions; (B) Predicting the potential for phase separation to occur at HNRNPC by PhaSePred (<http://predict.phasep.pro/>); (C) Construction of HNRNPC fluorescent plasmids with eGFP fused to the C-terminus of the protein; (D) Representative images and quantitative analysis of puncta in 231BO and 231 cells transfected with HNRNPC-eGFP treated with 10% 1,6-hexanediol (1,6-HD) for 10 min or not. n = 3. Scale bar, 10 µm; (E)Primary amino acid sequence of HNRNPC. Residues highlighted in red indicate the specific mutation sites within IDR1 and IDR2 for the generation of mutant plasmids.

**Figure S5: Expression of HNRNPC in breast cancer and its association with patient prognosis. (**A, B) UALCAN database analysis showing that both mRNA (A) and protein (B) levels of HNRNPC are significantly higher in BC tissues compared with adjacent normal tissues. (C) GSE2034 dataset analysis indicating that HNRNPC expression is markedly elevated in breast cancers with bone metastasis compared to other metastatic sites; (D, E) qPCR and Western blot analyses showing that HNRNPC expression is highest in the bone metastatic cell line 231-BO; (F) Representative immunohistochemical staining of HNRNPC in breast cancer specimens from FUSCC, including 42 non-metastatic breast cancers with paired adjacent normal tissues and 33 paired primary and bone metastatic tissues; (G) Kaplan–Meier Plotter database analysis showing that high HNRNPC expression is associated with poor overall survival in Luminal A, Luminal B, HER2+, and Basal-like breast cancer subtypes; (H, I)Loss-of-function and rescue assays in 231BO cells; knockdown of HNRNPC significantly inhibits cell migration, invasion, and proliferation, whereas re-expression of HNRNPC effectively restores these malignant phenotypes.

**Figure S6: HNRNPC specifically binds to and regulates IGF1R mRNA in breast cancer cells.** (A) Heatmap illustrating the differentially expressed genes (DEGs) in 231-BO cells following HNRNPC knockdown. (B) Cross-referencing of eCLIP-seq, RIP-seq, and RNA-seq data identifying IGF1R mRNA as a specific downstream target of HNRNPC. (C) Correlation analysis of HNRNPC and IGF1R mRNA expression levels based on TCGA databases. (D) Representative immunohistochemical (IHC) staining images demonstrating significantly elevated IGF1R protein levels in breast cancer bone metastasis tissues; (E): Rescue experiment showing that knockdown of IGF1R counteracts the pro-metastatic and osteoclastogenic effects induced by HNRNPC overexpression. Representative images (left) and quantification (right) of transwell migration, invasion, and TRAP-positive osteoclast formation assays in Control, OE-HC, and OE-HC + Si-IGF1R group in 231 cells. **Note: Representative images for Control and OE-HC groups are reused from Figure 6B and 6E (Main Figure) in comparison with OE-HC group**. HC: HNRNPC; (F) Pharmacological inhibition of ERK by HY-12275 suppresses cell migration and invasion. Representative images (left) and quantitative analysis (right) of transwell migration and invasion assays in cells treated with negative control (NC) or the ERK inhibitor (HY-12275). Data are presented as mean ± SD from three independent experiments (n = 3). *p < 0.05, **p < 0.01, ***p < 0.001 by one-way ANOVA test (E) or two-tailed unpaired t-test (F).

**Figure S7: Validation of HNRNPC expression, correlation with p-ERK, and prognostic value in clinical cohorts.** **(A)** Representative immunohistochemistry (IHC) images showing HNRNPC (HC) and p-ERK protein expression levels in bone metastasis tissues (high vs. low expression groups). Scale bar:100μm. Correlation analysis between HNRNPC IHC score and p-ERK IHC score in human bone metastasis lesions ($n=33$, Spearman rank correlation, 𝑅=0.37, 𝑃=0.035); (B) Receiver operating characteristic (ROC) curve showing the diagnostic performance of serum 5’tiRNA-Gln level in predicting bone metastasis (N = 75, AUC = 0.835, 95%CI: 0.747-0.924); (C) Forest plot displaying the multivariable Cox regression analysis of independent risk factors associated with overall survival, including Age, TNM stage, Tumor grade, Ki-67, and HNRNPC expression levels (HR = 2.845, 95CI: 1.554-5.207, P < 0.001).
