# Supplementary material for: Transfer RNA‐Derived Small RNA‐5’tiRNAGln Drives HNRNPC Phase Separation to Promote Breast Cancer Bone Metastasis by Regulating IGF1R
Source: Adv Sci (Weinh). 2026 Sep 29:e77974. Online ahead of print. doi: 10.1002/advs.77974 (PMC13624320; doi:10.1002/advs.77974)
Supplement: Supplementary file 2 [file ADVS-9999-e77974-s003.zip › Supplemental tables(S4-S8).docx]

**Supplementary Tables**

**Table S1. The sequencing analysis of tsRNA from 3 breast cancer and paired bone metastasis tissues**

**Table S2. The results of protein spectrum analysis from RNA-pull down assay**

**Table S3: Intersection of eCLIP-seq, RIP-seq, and RNA-seq datasets**

**Table S4. Antibodies manufacturer and catalog number**

**Table S5. Primers and probes for RT-qPCR assays**

**Table S6. Primers and probes for Rip-qPCR assays**

**Table S7. sequences of 5’tiRNA-Gln mimics, Inhibitors**

**Table S8. Sh-RNA and overexpression RNA-sequence**

**Table S9. Clinicopathological characteristics of the breast cancer cohorts**

Detailed clinical features of patients with paired primary breast tumors and bone metastases (Sheet 1), and primary breast tumors without bone metastasis (Sheet 2). Data include patient demographics, primary tumor size, lymphatic metastasis status, treatment history (surgery and neoadjuvant therapy), and the immunohistochemical expression levels of standard biomarkers (ER, PR, HER2, and Ki67) across both primary and metastatic lesions. Abbreviations: ER, estrogen receptor; PR, progesterone receptor; HER2, human epidermal growth factor receptor 2; BM, bone metastasis; PCR, pathological complete response. For immunohistochemistry results: “-”, negative; “+/++/+++”, varying intensities of positive expression.

**Table S4.**

| **Antibodies** | **Vendors** | **Catalog number** |
| --- | --- | --- |
| HNRNPC | Proteintech | 11760-1-AP |
| IGF1R | Proteintech | 20254-1-AP |
| Beta Actin | Proteintech | 66009-1-Ig |
| Histone H3 | Proteintech | 17168-1-AP |
| Flag tag | Cell Signaling Technology | 14793 |
| ERK | Cell Signaling Technology | 4695 |
| p-ERK | Cell Signaling Technology | 5726 |
| Goat anti-Rabbit IgG antibody (HRP) | Proteintech | No.SA00001-2 |
| Goat anti-Mouse IgG antibody (HRP) | Proteintech | No. SA00001-1 |

**Table S5.**

| **Gene** | **Primer and probe sequences** |
| --- | --- |
| **GAPDH** | forward:5’-GGTCACCAGGGCTGCTTTTA-3’  reverse:5’-GGATCTCGCTCCTGGAAGATG-3’ |
| **HNRNPC** | forward:5’-TGGGCTGCTCTGTTCATAAGGG-3’  reverse:5’- CTCGGTTCACTTTTGGCTCTGC-3’ |
| **IGF1R** | forward:5’- TCACCCAAAGCAGAAACTCA-3’  reverse:5’-CGGTGATGTTGTAGGTGTCTGC-3’ |

**Table S6.**

| **Gene** | **Primer and probe sequences** |
| --- | --- |
| **5’tiRNA35** | forward F: CATGGTGTAATGGTTAGCACTCTG  Universal Primer R:  5’-AGTGCAGGGTCCGAGGTAT-3’ |
| **IGF1R** | forward:5’-CCTGCACAACTCCATCTTCGTG-3’  reverse:5’-GGCCCCCTGACTACAGGGAT-3’ |

**Table S7.**

| **Gene** | **Primer and probe sequences** |
| --- | --- |
| **5’tiRNAGln mimics#1** | CATGGTGTAATGGTTAGCACTCTG |
| **Mimics NC** | UUUGUACUACACAAAAGUACUG |
| **5’tiRNAGln inhibitor#1** | CAGAGUGCUAACCAUUACACCAUG |
| **5’tiRNAGln inhibitor#2** | TCCAGAGTGCTAACCATT |
| **Inhibitor NC** | CAGUACUUUUGUGUAGUACAAA |

**Table S8.**

| **Gene** | **Primer and probe sequences** |
| --- | --- |
| **Sh-HNRNPC#1** | GCAGAGCAGCAGCTCCGTGAA |
| **Sh-HNRNPC#2** | GCGCCAATGGCGAGGATGACT |
